# Identification and functional analysis of *ZmDLS* associated with the response to biotic stress in maize

**DOI:** 10.3389/fpls.2023.1162826

**Published:** 2023-07-20

**Authors:** Yiting Wang, Jie Zou, Jiali Li, Fanna Kong, Lina Xu, Dafeng Xu, Jiaxin Li, Huaying Yang, Lin Zhang, Tingchun Li, Honghong Fan

**Affiliations:** ^1^ School of Life Science, Anhui Agricultural University, Hefei, China; ^2^ Tobacco Research Institute, Anhui Academy of Agricultural Sciences, Hefei, China; ^3^ Institute of Plant Protection and Agro-products Safety, Anhui Academy of Agricultural Sciences, Hefei, China

**Keywords:** maize, terpene synthase, corn borer bite, *Fusarium graminearum* attack, defense response, transcription factor

## Abstract

Terpenes are the main class of secondary metabolites produced in response to pest and germ attacks. In maize (*Zea mays* L.), they are the essential components of the herbivore-induced plant volatile mixture, which functioned as a direct or indirect defense against pest and germ attacks. In this study, 43 maize terpene synthase gene (*ZmTPS*) family members were systematically identified and analyzed through the whole genomes of maize. Nine genes, including *Zm00001d032230*, *Zm00001d045054*, *Zm00001d024486*, *Zm00001d004279*, *Zm00001d002351*, *Zm00001d002350*, *Zm00001d053916*, *Zm00001d015053*, and *Zm00001d015054*, were isolated for their differential expression pattern in leaves after corn borer (*Ostrinia nubilalis*) bite. Additionally, six genes (*Zm00001d045054*, *Zm00001d024486*, *Zm00001d002351*, *Zm00001d002350*, *Zm00001d015053*, and *Zm00001d015054*) were significantly upregulated in response to corn borer bite. Among them, *Zm00001d045054* was cloned. Heterologous expression and enzyme activity assays revealed that *Zm00001d045054* functioned as d-limonene synthase. It was renamed *ZmDLS*. Further analysis demonstrated that its expression was upregulated in response to corn borer bites and *Fusarium graminearum* attacks. The mutant of *ZmDLS* downregulated the expressions of *Zm00001d024486*, *Zm00001d002351*, *Zm00001d002350*, *Zm00001d015053*, and *Zm00001d015054*. It was more attractive to corn borer bites and more susceptible to *F. graminearum* infection. The yeast one-hybrid assay and dual-luciferase assay showed that ZmMYB76 and ZmMYB101 could upregulate the expression of *ZmDLS* by binding to the promoter region. This study may provide a theoretical basis for the functional analysis and transcriptional regulation of terpene synthase genes in crops.

## Introduction

Volatile terpenes are widely exist in plant tissues such as leaves, flowers, and fruits. They play critical roles in pollinator attraction, herbivore predator attraction, and predator repellent. They function as essential substances for plant growth, development, and disease resistance (Dudareva et al., 2005; Yu and Utsumi, 2009; Nagegowda, 2010; Murali-Baskaran et al., 2022).

The biosynthesis of terpenoid occurs via two pathways, namely the mevalonate pathway (MVA) in the cytoplasm and the methylerythritol 4-phosphate pathway (methylerythritol pathway (MEP)) located in the plastid (Cheng et al., 2007). The synthesis of terpenoids consists of three main parts: the synthesis of intermediate isopentenyl pyrophosphate (IPP) and allyl isomer dimethyl allyl pyrophosphate (DMAPP), the formation of precursor substances, including geranylgeranyl diphosphate (GPP), farnesyl diphosphate (FPP), and geranylgeranyl diphosphate (GGPP), and the generation of the terpenes (Vranova et al., 2013; Bergman et al., 2019). Terpene synthase (TPS) catalyzed the synthesis of the terpenes at the last step by using FPP, GPP, and GGPP as substrates.

Plant terpenoids play an important role in direct and indirect defense against biotic stress. In rice, OsTPS46 catalyzed the synthesis of constitutive limonene and (*E*)-β-farnesene, which may play a crucial role in protection against *Rhopalosiphum padi* (Sun et al., 2017). OsTPS19 functions as an (*S*)-limonene synthase. The products of OsTPS19 (*S*)-limonene could inhibit the growth of *Magnaporthe oryzae* spores *in vitro*, which protected the plants from the infection of rice blast disease (Chen et al., 2018). In maize, TPS10 products (*E*)-α-bergamotene and (*E*)-β-farnesene could be consistently induced by herbivory. In addition, overexpression of TPS10 in *Arabidopsis* generated a volatile signal that attracted *Cotesia marginiventris* (Köllner et al., 2009). Similarly, the emissions of constitutive volatiles from ZmTPS8 were attractive to *C. marginiventris* (Fontana et al., 2011). Further analysis revealed that ZmTPS8 is a multiproduct *α*-copaene synthase. Its product, cubebol, exhibited significant antifungal activity against both *Fusarium graminearum* and *Aspergillus parasiticus in vitro* (Saldivar et al., 2023). Otherwise, biosynthesis and emission of (*E*, *E*)-α-farnesene triggered by caterpillars and aphids at a high density functioned as a phytochemical signal, attracting their natural enemies, *Diadegma semiclausum* parasitoids in *Arabidopsis* (Kroes et al., 2017). In addition, three terpene alcohols farnesol, nerolidol, and plaunotol, are toxic and have antibacterial effects on *Staphylococcus aureus* (Inoue et al., 2004).

Recently, *TPS* genes and their families have been identified in the genomes of *Arabidopsis thaliana*, *Solanum lycopersicum*, and *Oryza sativa* (Aubourg et al., 2002; Chen et al., 2011; Falara et al., 2011). In maize, more than a dozen *TPS* genes have been isolated and analyzed (Shen et al., 2000; Ren et al., 2016; Block et al., 2019; Köllner et al., 2020). In this study, the *TPS* gene families were systematically identified and analyzed. The genes expressed explicitly in the leaf were tested in response to corn borer bite. One *TPS* gene named *ZmDLS* was cloned and functionally analyzed. These results may provide new insight into the vital role of *TPS* genes in plants’ responses to insect and pathogen attacks.

## Materials and methods

### Plant material

The maize-inbred line B73 and its *zmdls* mutant were obtained from the maize Ethyl methanesulfonate (EMS)-induced mutant database (http://www.elabcaas.cn/memd/public/index.html#/). The seedlings were cultivated in a greenhouse at 26°C with 16/8-h light/dark cycle. The leaves were collected at the eighth leaf stage for the following experiments.

### Identification of *TPS* genes from the maize genome

The hidden Markov model (HMM) file was generated using two TPS domains, PF03936 and PF01397, acquired from the Pfam database (http://pfam.xfam.org/). It was then used as a query to blast against the B73 RefGen v4 protein database (https://download.maizegdb.org/Zm-B73-REFERENCE-GRAMENE-4.0/) by HMMER v3.3.2 (http://www.hmmer.org/). The candidate sequences were validated using the SMART and NCBI CDD (http://smart.embl-heidelberg.de/). Finally, the ExPASy (http://web.expasy.org/protparam/) was employed to examine candidate sequences. Also, Wolfpsort (https://wolfpsort.hgc.jp/) was applied to predict the subcellular localization of *TPS* genes.

### The alignment and phylogenetic analysis of TPS proteins

Based on the Cluster W tool, the candidate sequences were aligned with TPS proteins from *Oryza sativa* and *Arabidopsis thaliana* (Aubourg et al., 2002; Chen et al., 2011). The neighbor-joining tree was constructed with a 1,000 bootstrap value using MGEA-X.

### Analysis of gene structure and recognition of conserved motifs

The TBtools (http://www.tbtools.com/) were employed to analyze the exon-intrinsic structure. The MEME software (http://meme-suite.org/) was used to predict the conservative pattern of ZmTPS as described by Bailey et al. (2009). The number of discovered motifs ranges from 1 to 10. The width of the motifs was set as greater than 6 and less than 200, respectively.

### Identification of *cis*-acting elements in ZmTPS promoter

A 2,000-bp upstream promoter sequence was downloaded for each *TPS* gene from the maize genome. The online tools PlantCARE (http://bioinformatics.psb.ugent.be/webtools/plantcare/html/) and New PLACE (https://www.dna.affrc.go.jp/PLACE/?action=newplace) were used to identify the *cis*-acting regulatory elements.

### Chromosomal distribution and genetic interactions of *TPS* gene

The chromosomal locus information for the *TPS* gene was obtained from the Maize Genetics and Genomics Database (https://www.maizegdb.org/) (Portwood et al., 2019). The physical locations and genetic interactions were predicted by TBtools software (Chen et al., 2020).

### Tissue-specific expression analysis of *ZmTPS* genes

To analyze the tissue-specific expression pattern of the ZmTPS family genes, raw data of the transcriptome of 23 different tissues (mature leaf, primary root, mature pollen, and silk, etc.) for B73 was downloaded from qTeller of maizeGDB (https://qteller.maizegdb.org/). Fragments per kilobase of transcript per million mapped read (FPKM) values were used to evaluate the relative transcription abundance. The TBtools were employed to draw the heat maps. The gradient color from blue to red indicates the log2 fold change value from FPKM data.

### Prokaryotic expression and enzyme activity assays

The constructed pMAL-c2x-*Zm00001d045054* recombinant plasmid was introduced into BL21(DE3) expression strains and screened for positive clones. Subsequently, 50 μL of the bacterial solution was aspirated with a pipette under aseptic conditions and added into 5 mL of Luria–Bertani (LB) medium containing ampicillin in a centrifuge tube. After incubating at 37 °C and shaking at 200 r/min overnight, 2 mL of turbid bacterial solution was added into 100 mL of LB medium containing the ampicillin and shaking at 200 r/min for 2–3 h at 37 °C until the bacterial solution reached an OD_600_ = 0.6–0.8.

Isopropyl β-d-thiogalactoside (IPTG) with a final concentration of 0.5 mM was used to induce the recombinant protein expression. The bacteria solution was induced overnight at 18 °C and then centrifuged at 11,000×*g* at 4 °C for 10 min. The precipitate was resuspended in a 3-mL buffer solution containing 20 mM Tris-HCl at pH 8.0, 10 mM dithiothreitol (DTT), 5 mM Na_2_S_2_O_5_, and 10 % glycerin to generate a crude enzyme solution after subsequent ultrasonication (five pulses of 20 s at 15 W, 4 °C) (Yahyaa et al., 2015b). The catalytic activity of recombinant *Zm00001d045054* was analyzed using geranyl diphosphate (GPP) as a substrate. Enzyme activity was determined in a headspace vial containing 500 µL of crude enzyme, 50 mM Tris-HCl at pH 7.5, 10 mM MgCl_2_, 10 µM MnCl_2_, and 10 µM GPP in a final total volume of 1 mL (Yahyaa et al., 2015a). The solid-phase microextraction (SPME) with a 100-µm polydimethylsiloxane (PDMS) fiber was then inserted into the headspace bottle. After extraction for 60 min at room temperature, the samples were analyzed using GC-MS to identify volatile terpenes (Li et al., 2021). The components of volatile terpenes were verified by comparing them with the National Institute of Standards and Technology (NIST) 2011 Standard Spectrum Library (https://www.nist.gov/srd/nist-standard-reference-database-1a).

### Identification of *ZmMYBs* associated with the control of *ZmDLS* expression

The raw data of transcriptomes of 23 different tissues for B73 were downloaded from qTeller of maizeGDB (https://qteller.maizegdb.org/). The FPKM values were used to evaluate the relative transcription abundance and construct the gene co-expression network for *ZmDLS* and the 157 *ZmMYBs* that have been reported (Du et al., 2012). The cor function in R language (https://www.r-project.org/) was used to calculate the *p*- and *r*-values of these gene pairs. The *ZmMYBs* with values *p* < 0.01 and *r* > 0.6 were selected for further analysis.

### Yeast one hybrid assay

The *ZmDLS* promoter *cis*-acting elements were predicted using the online tools PlantCARE (http://bioinformatics.psb.ugent.be/webtools/plantcare/html/) and New PLACE (https://www.dna.affrc.go.jp/PLACE/?action=newplace) (Lescot et al., 2002).

The *ZmDLS* promoter fragment and the full-length ZmMYBs were cloned into pAbAi and pGADT7 vectors, and then the pAbAi-*ZmDLS* vector was linearized and integrated into the Y1HGold yeast genome. The plasmid pGADT7-*ZmMYBs* were transformed into decoy strains, screened for positive clones, and coated in SD/-Leu solid medium containing 100 ng·mL^−1^ Aureobasidin A (AbA) (**Supplementary Figure S5**) (Li et al., 2017). Three biological replicates were created in the experiment.

### Subcellular localization of ZmDLS and ZmMYBs

The full-length CDS of *ZmDLS* was cloned into the pCAMBIA1305 vector, forming a fusion construct pCAMBIA1305-*ZmDLS*-GFP. Then, it was transformed into *Agrobacterium tumefaciens* strain GV3101 and subsequently inoculated in the leaves of 4-week-old *Nicotiana tabacum* as described by Wang et al. (2021). The empty vector was used as a negative control. In detail, the leaves of *Nicotiana tabacum* were cut into about 1.0-mm-long strips and then soaked in the enzymatic digestion solution (**Supplementary Table S6**). To digest the cell walls, the incubation was conducted at room temperature by shaking at 40 rpm/min for 5–7 h. After filtering with a 100-mesh cell strainer, the tube containing the solution was centrifuged at 11,000×*g* at 4 °C for 10 min. The precipitate was washed with a W5 solution. Finally, the protoplasts were re-suspended in MMg solution (100 μL) and observed under laser confocal microscopy.

Similarly, fusion constructs pCAMBIA1305-*ZmMYB*-GFP were transformed into *Agrobacterium tumefaciens* strain GV3101. Then, it was co-cultured with 1 cm^2^ fresh onions epidermis at 22 °C for 20 h in the dark in the co-culture solid medium (½ MS, 1 % sucrose, 0.03 % casein, 0.28 % proline, 10 μmol/L 2,4-d, 2 μmol/L 6-BA, 200 μmol/L As, and 0.8 % agar) (Thakur et al., 2021). The epidermis was observed and documented under laser confocal microscopy.

### Dual-luciferase assay

Approximately 1,200 bp of the *ZmDLS* promoter were cloned into the pGreenII-0800-LUC vector to create the pZmDLSpro : LUC reporter construct. The full-length cDNAs of ZmMYB76 and ZmMYB101 were cloned into the pGreenII 62-SK vector. The sequences of all primers are provided in **Supplementary Table S5**. The constructs were introduced into *Agrobacterium* GV3101 and then co-transformed into the leaves of *Nicotiana tabacum*. One-half part of the leaf was injected with pGreenII 0800-*ZmDLSpro*-LUC plus pGreenII 62-SK-ZmMYB infestation solution, and the other part was injected with pGreenII 0800-*ZmDLSpro*-LUC bacterial solution as the control. The enzymatic activities of firefly and Renilla luciferases were measured with a multifunctional enzyme labeler (Thermo Fisher, Carlsbad, California, USA) using the Dual‐Luciferase Assay Kit (Beyotime, Shanghai, China). The luciferase activity was calculated as described by Yeasn’s instructions.

### RNA isolation and qRT-PCR analysis

The maize inbred line B73 and *zmdls* mutant leaves were collected at the eight-leaf stage. Total RNA was extracted with the RNA easy Plant Kit (Best Technology, Beijing, China) and then reverse transcribed to cDNA using MonScript™ RTIII Super Mix with dsDNase (Monad, Suzhou, Jiangsu, China). The qRT-PCR reaction system contained 50 ng of cDNA, 0.2 μM of forward primer, 0.2 μM of reverse primer, and 1× Super Specificity qPCR Mix with a final volume of 20 μL. qRT-PCR analysis was conducted on a Real-time PCR detection system (Bio-Rad, Hercules, California, USA) using SYBR^®^ Green qPCR mix (MonScript, Monad, Suzhou, Jiangsu, China) following Li’s method (Li Z et al., 2020). The one-way ANOVA was used to estimate the significant differences between control and treatment based on the Tukey method.

### Insect feeding experiment

Four leaf discs with a diameter of 2 cm from the *zmdls* mutant and the wild type were placed on Petri dishes. In total, 20 samples of second-instar larvae of corn borer were transferred to the center of the dish in a chamber at 27 °C, relative humidity 60 %, and light duration 16/8-h light/dark cycle. According to Cao’s method, the percentage of surface area was calculated at 3, 6, 9, and 12 h (Cao et al., 2022). Otherwise, 30 third-instar larvae were fed with leaves from the *zmdls* mutant and wild type. After 4 days of feeding, each larva was weighed.

### *In vitro* antibacterial assay of d-limonene

The antibacterial activity of d-limonene was determined using the mycelial growth rate method (Li J. et al., 2022). The *F. graminearum* was placed on PDA mediums containing 0, 100, 200, and 400 μg·L^−1^ d-limonene, respectively. After incubating at 28 °C for 7 days, the mycelium in control was covered all over the petri dish, and the diameter of the colony was measured to calculate the inhibition rate as described by Zhang et al. (2021). To detect the infection of *F. graminearum* to the leaves of maize, the *F. graminearum* was cultured with a synthetic low-nutrient medium (SNA, 7.35 mM KH_2_PO_4_, 6.71 mM KCl, 9.89 mM KNO_3_, 1.11 mM Glucose, 0.58 mM Sucrose, 2.03 mM MgSO_4_.7H_2_O, 3.17 mM Agar) and incubated at 28 °C for 5–7 days until the concentration accounted for 10^6^ spores·mL^−1^. According to Cao’s method, the leaves were inoculated with spore suspension (Cao et al., 2022). Every treatment group contained four leaves and was incubated at 28 °C with 60 % humidity. The infected area of the leaves was calculated and documented with a camera at 3-6 d of incubation.

## Results

### Identification of the *TPS* gene family in maize

**Figure 1:**
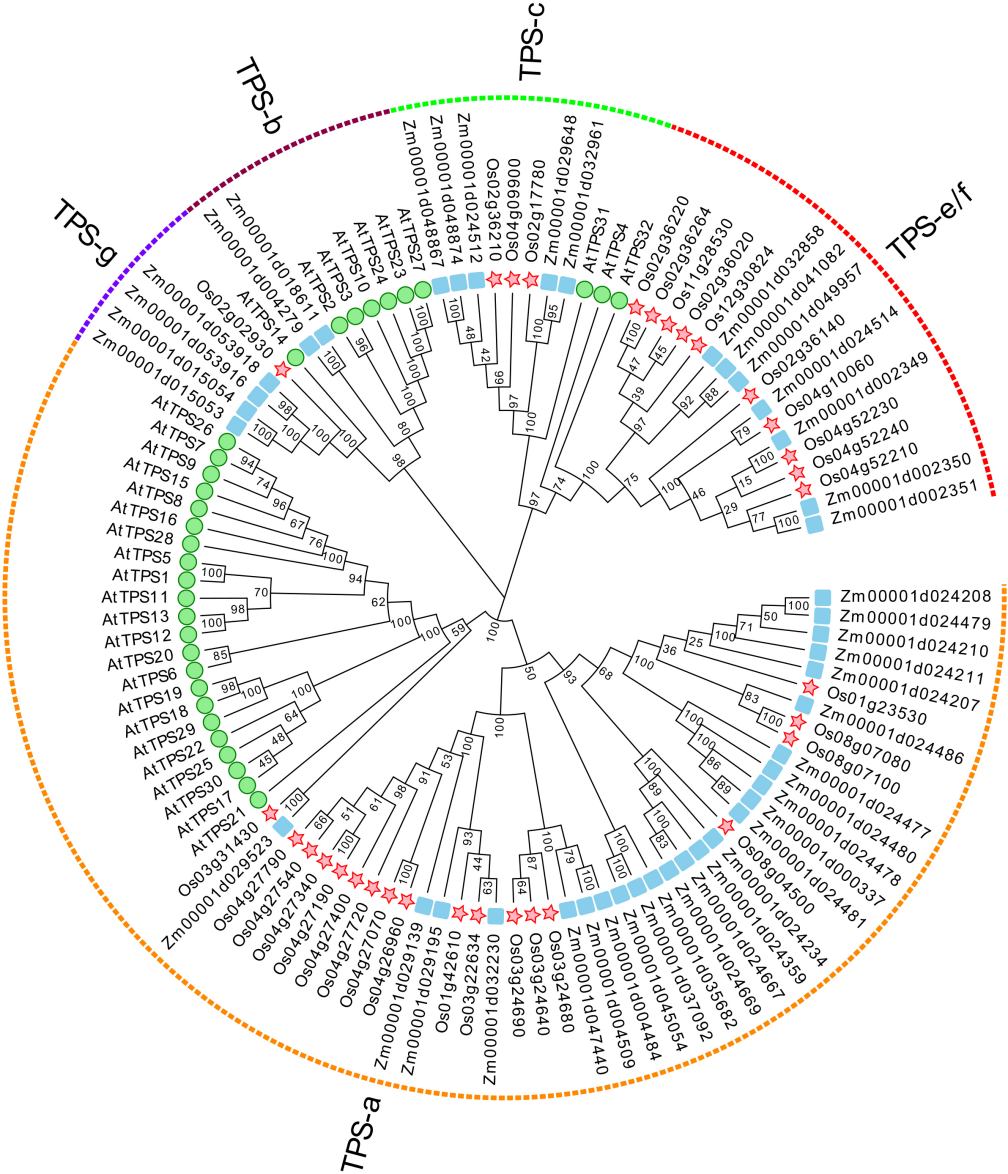
The phylogenetic analysis of ZmTPS proteins. The TPS proteins in *Z. mays*, *A. thaliana*, and *O. sativa* were used. The green circle represents the *TPS* genes of *A. thaliana*, the red pentagram represents the *TPS* genes of *O. sativa*, and the blue square represents the *TPS* genes of *Z. mays*. The neighbor-joining tree was drawn using the MEGA-X. The *TPS* gene family was divided into five subfamilies, designated TPS a–TPS f.

To systematically identify *TPS* genes in maize, a hidden Markov model (HMM) profile of the conserved C-terminal (PF03936) and N-terminal (PF01397) domains in TPS proteins was used as a root file to blast against the maize genome database (B73 RefGen v4 protein database). Significant hits with an e-value of < 10^−5^ were retrieved as candidate TPS proteins. The SMART and NCBI CDD online tools were used to verify the *TPS* genes. As shown in **Figure 1**, 43 sequences were identified and annotated as *ZmTPS* genes directly represented by the gene IDs (**Supplementary Table S1**). Further analysis revealed that the amino acid sequences of ZmTPSs ranged from 121 to 855 aa. The molecular weights were between 14.27 and 97.57 kDa. The isoelectric point changed from 4.78 to 7.47. The grand average of hydropathicity varied from 0.059 to −0.403, which showed that most of the ZmTPSs are hydrophilic proteins (**Supplementary Table S3**). Based on phylogenetic analysis, 43 *ZmTPS* genes were unevenly distributed in five TPS subfamilies. Among them, 25 *ZmTPS* genes were classified in the TPS-a subfamily, 2 *ZmTPS* genes belonged to the TPS-b subfamily, 5 *ZmTPS* genes were grouped into the TPS-c subfamily, 7 *ZmTPS* genes were categorized into the TPS-e/f subfamily, and 4 *ZmTPS* genes were classified into the TPS-g subfamily (**Figure 1**). The members from the same subfamily contained the same conserved motifs and a similar exon–intron structure (**Supplementary Figure S1**). Chromosomal localization and microsynteny analysis showed that most *ZmTPS* genes were located on chromosomes 10, 2, 1, and 4 (**Supplementary Figure S2**). Co-linearity analysis elucidated the relationship between the 43 *ZmTPSs*. As shown in **Supplementary Figure S3**, a total of two collinear gene pairs were identified in the maize genome. Among them, *Zm00001d037092* on chromosome 6 is co-linear with *Zm00001d045054* on chromosome 9. Also, *Zm00001d053916* on chromosome 4 is co-linear with *Zm00001d015054* on chromosome 5. Otherwise, the identification of *cis*-elements, including the abscisic acid responsiveness element (ABRE), MeJA-responsive motifs TGACG, gibberellin-responsive motifs, and the stress-related component of the promoter region of *ZmTPS* genes, predicted the possible role of *ZmTPS* genes in plant growth, development, and stress response (**Supplementary Figure S4**).

### Identification of tissue-specific expressed *ZmTPS* genes in maize leaf

**Figure 2:**
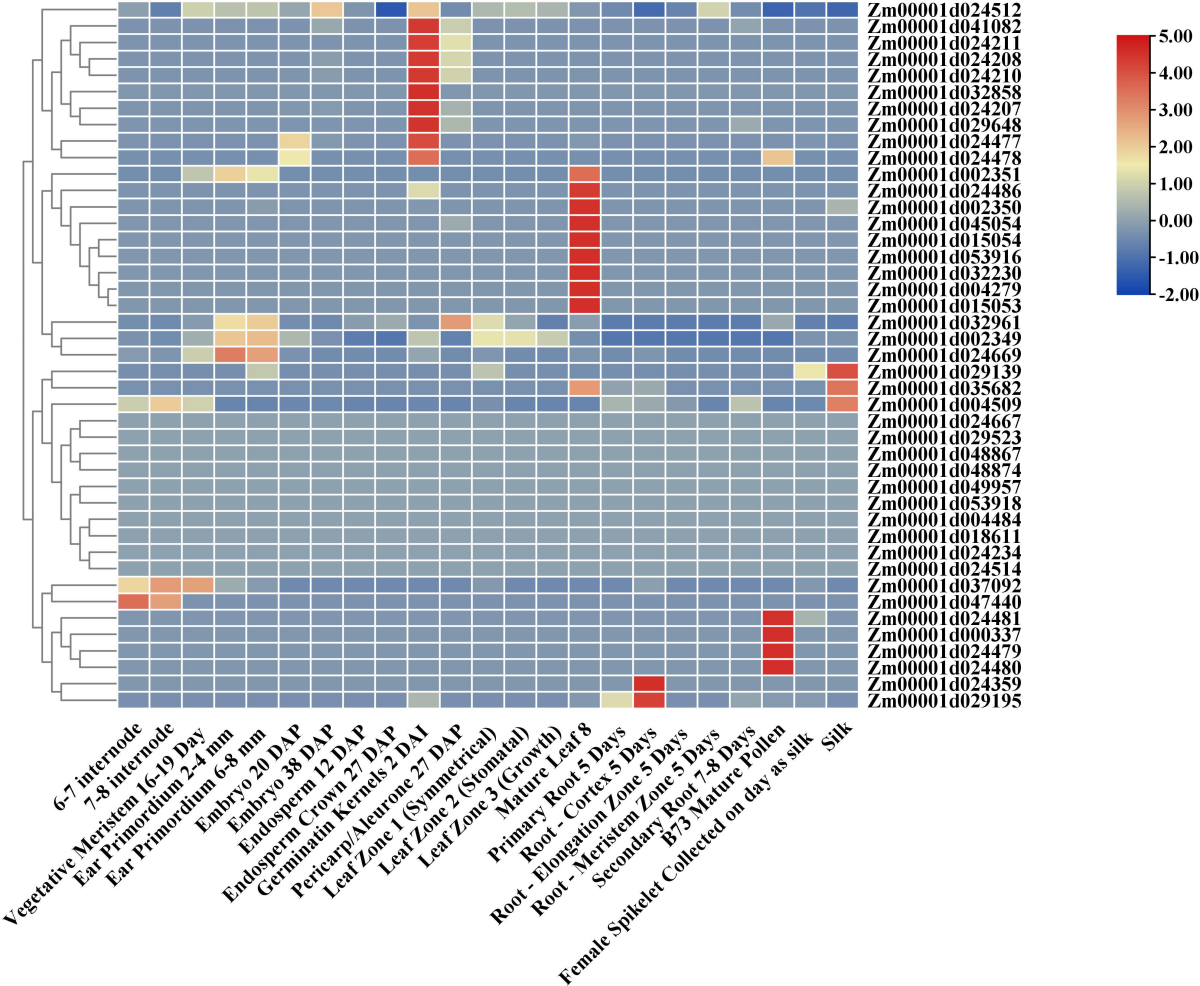
Expression profiles of 43 *ZmTPS*s in different tissues of maize. The raw data of the transcriptome of 23 different tissues for B73 were downloaded from qTeller of maizeGDB. FPKM values were used to evaluate the relative transcription abundance. The gradient color from blue to red indicates the log2 fold change value from FPKM data.

As shown in **Figure 2**, the expression patterns of *ZmTPS* genes in 23 tissues of maize were analyzed. The results exhibited a distinct tissue-specific expression pattern for 43 *ZmTPS* genes. Among them, *Zm00001d041082*, *Zm00001d024211*, *Zm00001d024208*, *Zm00001d024210*, *Zm00001d032858*, *Zm00001d024207*, *Zm00001d029648*, *Zm00001d024477*, and *Zm00001d024478* displayed the highest expression level in the germination of kernels. *Zm00001d024481*, *Zm00001d000337*, *Zm00001d024479*, and *Zm00001d024480* presented high expression levels in mature pollen. *Zm00001d029139*, *Zm00001d035682*, and *Zm00001d004509* showed high expression levels in silk. *Zm00001d024359* and *Zm00001d029195* displayed the highest expression level in root-cortex. *Zm00001d002351*, *Zm00001d024486*, *Zm00001d002350*, *Zm00001d045054*, *Zm00001d015054*, *Zm00001d053916*, *Zm00001d032230*, *Zm00001d004279*, and *Zm00001d015053* were found only highly expressed in mature leaf. *Zm00001d024512*, *Zm00001d032961*, *Zm00001d002349*, *Zm00001d024669*, *Zm00001d037092*, and *Zm00001d047440* expressed explicitly in other tissues.

### Identification of *ZmDLS* in response to the maize borer’s bite in the leaf

**Figure 3:**
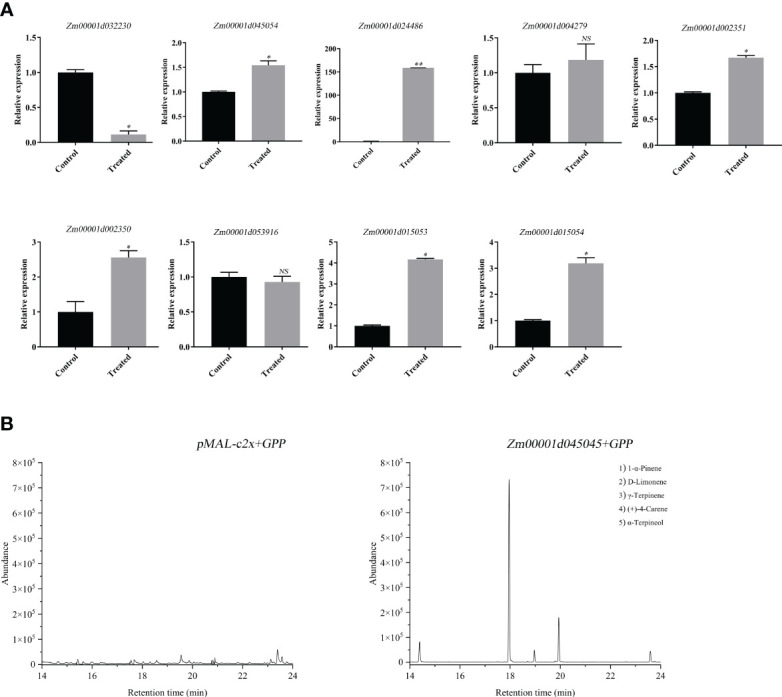
Functional analysis of *ZmTPS* genes in response to the maize borer’s bite. **(A)** Expression of *ZmTPS* genes after maize borer’s bite of the maize inbred line B73. Bars represent the mean values ( ± SD) of three biological replicates. Significance level (^*^ *p* < 0.05, **p < 0.01); NS means not significant (*p* > 0.05). **(B)** Headspace solid-phase microextraction combined with gas chromatography–mass spectrometry (HS-SPME-GC-MS) analysis of products generated *in vitro* by recombinant ZmDLS protein. GC-MS peaks of volatile compounds were detected using extracts of pMAL-c2x cells (left). GC-MS peaks of volatile compounds were detected using extracts of pMAL-c2x-Zm00001d045054 cells with the addition of GPP (right).

To study the role of the *ZmTPS* genes in the leaf, the expression of nine genes, including *Zm00001d032230*, *Zm00001d045054*, *Zm00001d024486*, *Zm00001d004279*, *Zm00001d002351*, *Zm00001d002350*, *Zm00001d053916*, *Zm00001d015053*, and *Zm00001d015054* were determined after the bite by the maize borer (*O.nabilalis*). The results showed that the expressions of six genes, including *Zm00001d045054*, *Zm00001d024486*, *Zm00001d002351*, *Zm00001d002350*, *Zm00001d015053*, and *Zm00001d015054* were significantly upregulated (**Figure 3A**). The expression of gene *Zm00001d032230* was downregulated. To further uncover the function of *ZmTPS* genes in response to the maize borer’s bite, the *Zm00001d045054* was cloned. Heterologous expression and enzyme activity assays of the *Zm00001d045054* in *Escherichia coli* showed that only one terpenoid, d-limonene, was identified in the enzymatic reaction products. The gene specifically catalyzed the synthesis of d-limonene using GPP. So, the gene *Zm00001d045054* was named *ZmDLS* according to the systematic nomenclature (**Figure 3B**).

### Acquisition and identification of *zmdls* mutants

**Figure 4:**
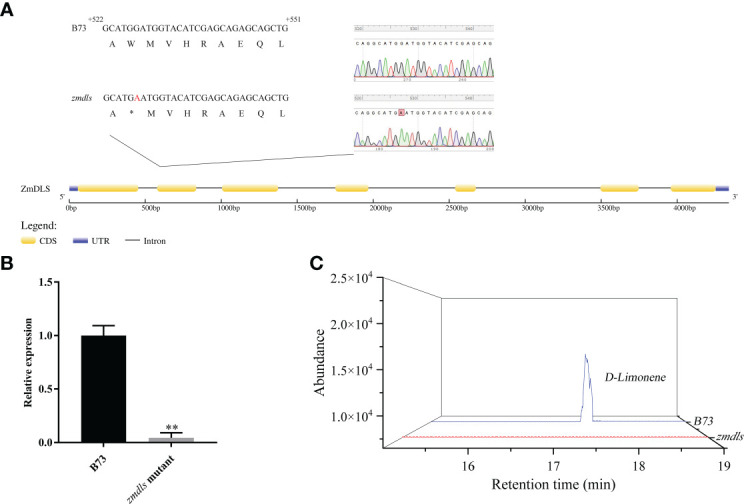
Acquisition and identification of *zmdls* mutants. **(A)** Sequence diagram of *ZmDLS* gene in the maize inbred line B73 and the *zmdls* mutant. **(B)** Expression of *ZmDLS* in the maize inbred line B73 and *zmdls* mutant. Bars represent the mean values ( ± SD) of three biological replicates. ^**^ *p* < 0.01, significant difference. **(C)** GC-MS analysis of d-limonene in maize inbred lines B73 and *zmdls* mutants.

To verify the molecular function of the *ZmDLS* gene, a B73 EMS-mutagenized mutant *zmdls* was obtained through the maize mutant website (www.elabcaas.cn/memd/). The mutation site was confirmed by sequencing the fragment obtained using PCR amplification. The results showed that the *zmdls* mutant has a mutation from G to A at 6 bp in the second exon region, which caused premature translation termination at 135 aa and the deletion of the DDXXD structural domain (**Figure 4A**).

Further experiments determined the content of d-limonene and the expression level of *ZmDLS* in the *zmdls* mutant. As shown in **Figures 4B, C**, the content of d-limonene was lowered, and the expression level of *ZmDLS* was significantly decreased in the *zmdls* mutant if compared with that in wildtype B73, which proved that the *zmdls* mutant was reliable and could be used for subsequent experiments.

### Functional analysis of *ZmDLS* gene

**Figure 5:**
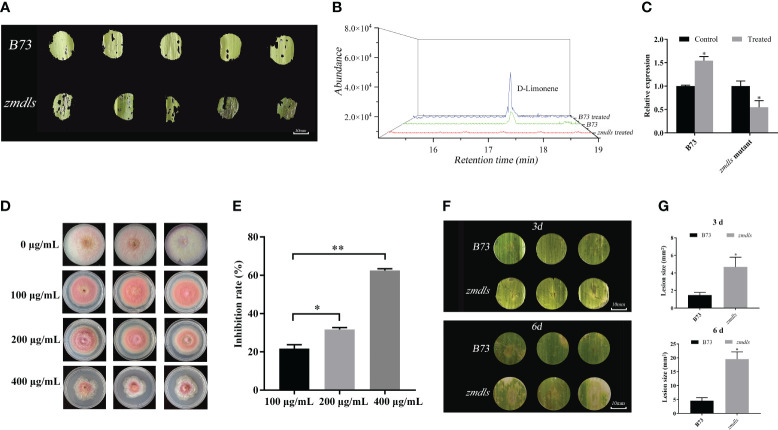
Functional analysis of *ZmDLS* in response to insect *O. nubilalis* and fungi *F. graminearum* attack. **(A)** The changes of a leaf of wild type and *zmdls* mutants after maize borer’s bite; each group contains at least three leaves. **(B)** GC-MS analysis of d-limonene in B73 and *zmdls* mutants after maize borer’s bite. **(C)** Expression of *ZmDLS* after maize borer’s bite in the maize inbred line B73 and *zmdls* mutants. Bars represent the mean values (± SD) of three biological replicates. ^*^ *p* < 0.05, significant difference. **(D)** *In vitro* antibacterial assay of d-limonene. Growth of *F. graminearum* in PDA medium at final concentrations of 0, 100, 200, and 400 μg/mL d-limonene. **(E)** *In vitro* antibacterial assay of d-limonene. There are three replicates in each group. *p < 0.05 and **p < 0.01, significant difference. **(F)** Wild type and *zmdls* mutants were inoculated with *F. graminearum in vitro*. Photographs were taken and the lesion area was measured at 3 and 6 days after inoculation. **(G)** *In vitro* inoculation of wild type and *zmdls* mutants with *F. graminearum* for 3 or 6 days for disease spot area. Each group contains at least three leaves. ^*^ *p* < 0.05, significant difference.

As is shown in **Figure 5A** and **Supplementary Figure S6**, the *zmdls* mutant was more alluring to larvae than the wild type when it was used to feed corn borer (*Ostrinia nubilalis*). The gene expression analysis revealed that the expression of *ZmDLS* was significantly downregulated in the mutant (**Figures 5B, C**). Otherwise, the expressions of *Zm00001d002351* and *Zm00001d002350* were downregulated too. The expression levels of *Zm00001d024486*, *Zm00001d053916*, *Zm00001d015053*, and *Zm00001d015054* were reduced after the bite if compared with that in the wild type (**Supplementary Figure S7**). The mutant of *ZmDLS* affected the other *ZmTPS* genes’ expressions in the leaf.

In addition, the *ZmDLS* product d-limonene was used to analyze its effects on the fungus *F. graminearum*. As is shown in **Figure 5D**, 100 μg·mL^−1^ of d-limonene inhibited the growth of *F. graminearum*. The higher concentration of d-limonene had a better inhibitory effect on *F. graminearum* (**Figure 5E**). *In vitro* culture of *F. graminearum* with the leaves of *zmdls* mutants showed that the mutant developed more significant necrotic lesions than wild-type leaves (**Figures 5F, G**).

### Analysis of *cis*-acting elements for *ZmDLS* promoter

**Figure 6:**
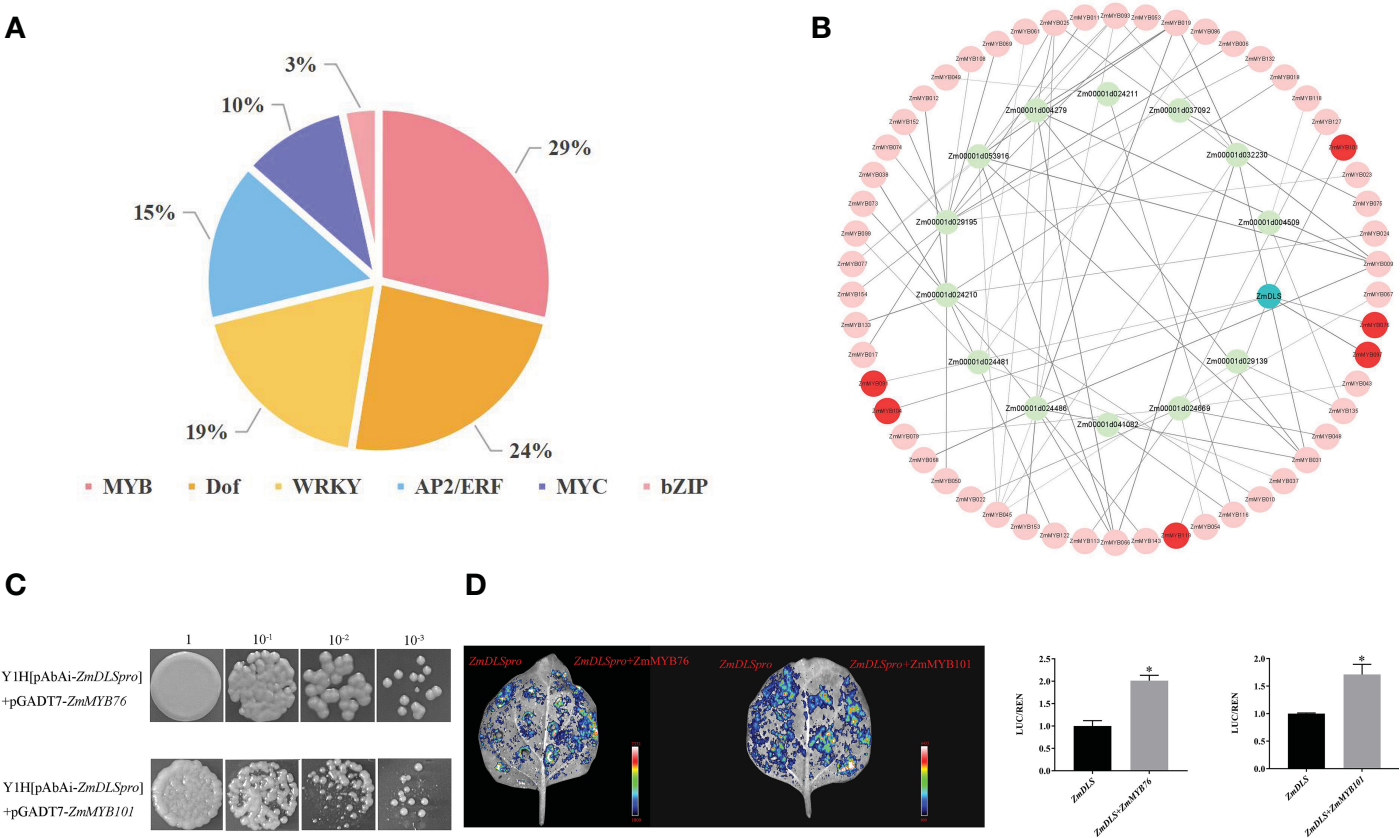
Interaction of *ZmMYB*s with *ZmDLS*. **(A)** *ZmDLS* promoter *cis*-acting site. Analysis of the 2,000 bp *ZmDLS* promoter and counting of the number of six transcription factor families contained in the *ZmDLS* promoter. **(B)** Co-expression network diagram of terpenoid synthesis-related genes and ZmMYB transcription factors. **(C)** Yeast single hybridization analysis of two transcription factors and *ZmDLS* promoters. Interactions were determined in the presence of AbA (AbA 100 ng/mL) on SD medium lacking leucine. **(D)** Co-expression of ZmMYB76 and ZmMYB101 with LUC driven by the *ZmDLS* promoters in the leaves of *N. tabacum*. An empty vector of pGreenII 62-SK was used as a control. Error bars, mean ± SD of three biological replicates. ^*^ *p* < 0.05, significant difference.

To identify the *ZmDLS* promoter, the 2,000-bp upstream region of *ZmDLS* was analyzed. The result showed that the promoter region was rich in *cis*-acting elements. For example, TGA motif, GARE motif, and ABRE belonged to phytohormone regulatory elements. The WRE3 motif was related to a damage induction element. TCA is a low-temperature regulatory element. The MBS was correlated with the drought response element. GCN4_motif and O_2_ site were grouped into growth and developmental regulatory elements. Further analysis of the *ZmDLS* promoter revealed that it mainly contains six typical plant transcription factor families, including MYB, MYC, WRKY, AP2/ERF, bZIP, and Dof (**Figure 6A**). However, the most enriched element is composed of several MYB binding sites, which possibly conferred the regulation role of MYB transcription factors on *ZmDLS*.

### *In vivo* binding of *ZmDLS* promoter to ZmMYBs

As shown in **Figure 6B**, 157 R2R3-MYB genes (named ZmMYB1-157) downloaded from the maize genome database were used to conduct co-expression network analysis with *ZmTPS*s. Six genes, including *ZmMYB97*, *ZmMYB76*, *ZmMYB101*, *ZmMYB104*, *ZmMYB119*, and *ZmMYB91*, were selected for their high correlation coefficients with *ZmDLS* (**Supplementary Table S7**). They were positively correlated with *ZmDLS*. Gene expression analysis showed that the expression of *ZmMYB76* and *ZmMYB101* was significantly upregulated after the bite by the maize borer, which was consistent with the expression of *ZmDLS* (**Figure 3A**; **Supplementary Figure S8**). The yeast one-hybrid (Y1H) assay showed that the *ZmDLS* promoter could be directly bound by ZmMYB76 and ZmMYB101 (**Figure 6C**). Also, further transient expression assays by agroinfiltration of the leaves of *N. tabacum* confirmed that the co-expression of LUC driven by ZmMYB76 or ZmMYB101 with *ZmDLS* promoters significantly increased the LUC/REN ratio, which indicated that ZmMYB76 and ZmMYB101 could upregulate the expression of *ZmDLS* (**Figure 6D**).

### Subcellular localization of ZmDLS and its regulatory genes in heterologous plants

**Figure 7:**
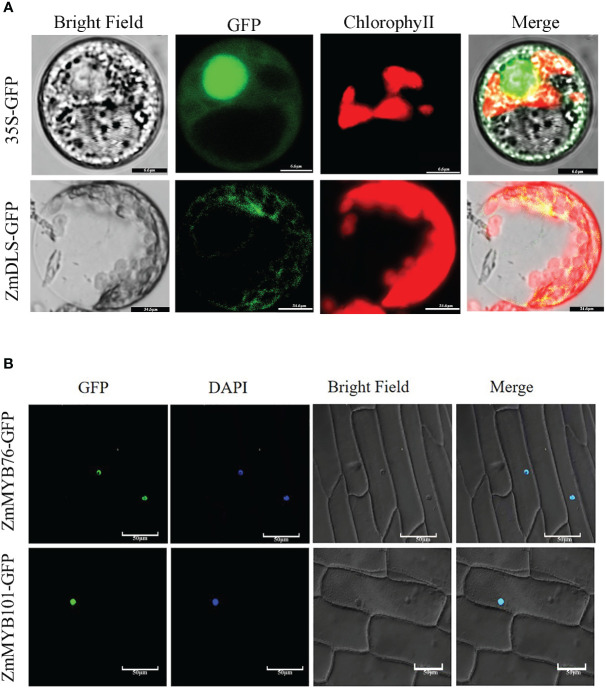
Subcellular localization of ZmDLS and ZmMYBs. **(A)** Subcellular localization of ZmDLS in the protoplast of *Nicotiana tabacum*. GFP is used as a control. **(B)** Subcellular localization of the ZmMYB-GFP fusion protein.

To investigate the function of *ZmDLS*, *ZmMYB76*, and *ZmMYB101* in maize, ZmDLS-GFP and ZmMYB-GFP fusion protein were transiently expressed in the leaves of *N. tabacum* and observed by laser confocal microscopy using a 35S-*GFP* vector as a control (**Figure 7A**). The green fluorescent fused ZmDLS-GFP gene was specifically distributed in chloroplasts. The result speculated that the gene might be involved in the MEP pathway and closely related to monoterpene synthesis. Nevertheless, ZmMYB-GFP only displayed a fluorescence signal in the nucleus, indicating that ZmMYB76 and ZmMYB101 are nuclear localization proteins (**Figure 7B**).

## Discussion

Maize is an important food crop and industrial raw material around the world. However, maize is infested with pests and germs, which will cause a large-scale reduction in yields. Terpenoids play a vital role in protecting plants from herbivores and microbial pathogens (Mafu et al., 2018; Block et al., 2019). Many maize terpene synthase genes have been studied previously, but a systematic analysis of the maize *TPS* gene family has been rarely reported (Ding et al., 2020). In this study, 43 *ZmTPS* genes were identified and systematically analyzed. These genes were unevenly distributed into TPS-a, TPS-b, TPS-c, TPS-e/f, and TPS-g subfamilies.

d-Limonene (C_10_H_16_) is a monocyclic monoterpene commonly found in citrus plants such as lemons, grapes, and oranges (Anandakumar et al., 2021). It was proven to be the chemical composition of the odor with the activities of antioxidants, antibacterials, and anti-insects (Geraci et al., 2017; Yu et al., 2017; Sanei-Dehkordi et al., 2022). DMNT, (*E*)-β-farnesene, and linalool could attract parasitic wasps or natural enemies, indirectly defending against pests. *S*-Linalool and (*E*)-β-caryophyllene have a tendency avoidance effect on brown rice planthopper *Nilaparvata lugens* (Xiao et al., 2012). In the experiment, ZmDLS catalyzes the synthesis of d-limonene. Further analysis demonstrated that d-limonene inhibited the growth of *F. graminearum*. The results were in accord with the previous research.

Many terpene synthase (TPS) genes have been identified to study the role of their product in protecting plants from pest and germ attacks. For example, overexpressing *GhTPS1* in cotton plants is less attractive to *Helicoverpa armigera*, *Apolygus lucorum*, and *Aphis gossipii* (Zhang et al., 2020). The decreased terpene content in tomato mutant leaves knocked out by *SlJIG* was more susceptible to *H. armigera*. The *jig-1* and *jig-16* mutant leaves produced more necrotic disease *in vitro* with *Botrytis cinerea* spores than wild-type leaves (Cao et al., 2022). Overexpression of *StTPS18* in potatoes made the plant more susceptible to the bacterial pathogen *P. syringae*. The tolerance to the *Ralstonia solanacearum* was enhanced (Dwivedi et al., 2022). Similarly, overexpression *of OsTPS19* in rice plants increased resistance to *M. oryzae*, whereas those with *OsTPS19* RNAi had increased susceptibility to this disease (Chen et al., 2018). In the experiment, the expression level of nine *TPS* genes *Zm00001d032230*, *Zm00001d045054*, *Zm00001d024486*, *Zm00001d004279*, *Zm00001d002351*, *Zm00001d002350*, *Zm00001d053916*, *Zm00001d015053*, and *Zm00001d015054* was determined after maize borer’s bite of the leaf. Six genes, including *ZmDLS*, were significantly upregulated in response to corn borer bite. The mutant *zmdls* downregulated the expressions of *Zm00001d024486*, *Zm00001d002351*, *Zm00001d002350*, *Zm00001d015053*, and *Zm00001d015054.* It was more attractive to corn borer bite and susceptible to *F. graminearum* infection.

Previous studies demonstrated that many kinds of transcription factors, including WRKY, AP2/ERF, and MYB, directly participated in the terpenes biosynthesis by regulating the expression of *TPS* genes (Xu et al., 2004; Yu et al., 2012; Sun et al., 2020; Lv et al., 2022). They bind to the *TPS* gene’s promoter region and positively or negatively regulate terpene biosynthesis (Vom Endt et al., 2002; Cao et al., 2020). For example, *CitTPS16* catalyzes the synthesis of geraniol in sweet orange. The transcription factor *CitERF71* can activate the expression of *CitTPS16* and enhance the accumulation of geraniol synthesis (Li et al., 2017). The transcription factor *AaMYC2* can bind to the G-box of *CYP71AV1* and *DBR2* promoters and regulate artemisinin synthesis in *Artemisia annua* (Shen et al., 2016). Otherwise, two transcription factors, *FhMYB21L1* and *FhMYB21L2*, can positively regulate *FhTPS1* expression. At the same time, *FhMYC2* can interact with *FhMYB21Ls* to form a blocker to inhibit the binding of *FhMYB21Ls* to the *FhTPS1* promoter, which negatively regulates the expression of *FhTPS1* (Yang et al., 2020). Previous studies demonstrated that MYB transcription factors regulate terpenoid biosynthesis in plants (Bedon et al., 2010; Shin et al., 2016; Li P. et al., 2020; Li H. et al., 2022; Lv et al., 2022). Two MYB transcription factor genes, FhMYB21L1 and FhMYB21L2, were synchronously expressed with *FhTPS1*. Overexpression of FhMYB21L1 and FhMYB21L2 could significantly upregulate the expression of *FhTPS1* in *the Freesia hybrid* (Yang et al., 2020). In *Hedychium coronarium* flowers, the R2R3-MYB transcription factor HcMYB2 could upregulate the expression of the linalool synthase gene HcTPS5 and enhance the emission of volatile compounds (Ke et al., 2021 *)*. In the experiment, two MYB transcription factors, ZmMYB76 and ZmMYB101, were verified to play an essential role in the upregulation of the expression of *ZmDLS* by binding to the region of the promoters, which directly increased the accumulation of d-limonene.

## Conclusion

In this study, the *TPS* gene family was systematically identified and analyzed through maize genomes. A monoterpene synthase gene, *ZmDLS*, was identified in response to a corn borer bite. ZmDLS was localized in the chloroplast and responsible for the biosynthesis of d-limonene, the chemical component of the odor, with the activities of antioxidant, antibacterial, and anti-insect. In addition, ZmMYB76 and ZmMYB101 could positively regulate the expression of *ZmDLS*. Otherwise, the experiment demonstrated that *ZmDLS* participated in the response against the insect *Ostrinia nubilalis* and the fungus *F. graminearum*. These findings could supplement a theoretical foundation for the functional study and transcriptional control of *TPS* genes in crops.

## Data availability statement

The original contributions presented in the study are included in the article/**Supplementary Material**. Further inquiries can be directed to the corresponding authors.

## Author contributions

TL and HF designed the experiment. YW and JZ conducted the experiment. JL, FK and LX helped with phenotypic analysis after the corn borer bite. DX, JXL, HY, and LZ were responsible for cultivating seedlings. All authors have read and approved the final manuscript.

## Supplementary material

Supplementary Table 8 Summary of qRT-PCR on data
