# Supplementary material for: Identification and functional analysis of ZmDLS associated with the response to biotic stress in maize
Source: Front Plant Sci. 2023 Jul 20;14:1162826. doi: 10.3389/fpls.2023.1162826 (PMC10399692; doi:10.3389/fpls.2023.1162826)
Supplement: Supplementary Table 8 — Summary of qRT-PCR on data [file DataSheet_1.zip › Supplementary Figures.PDF]

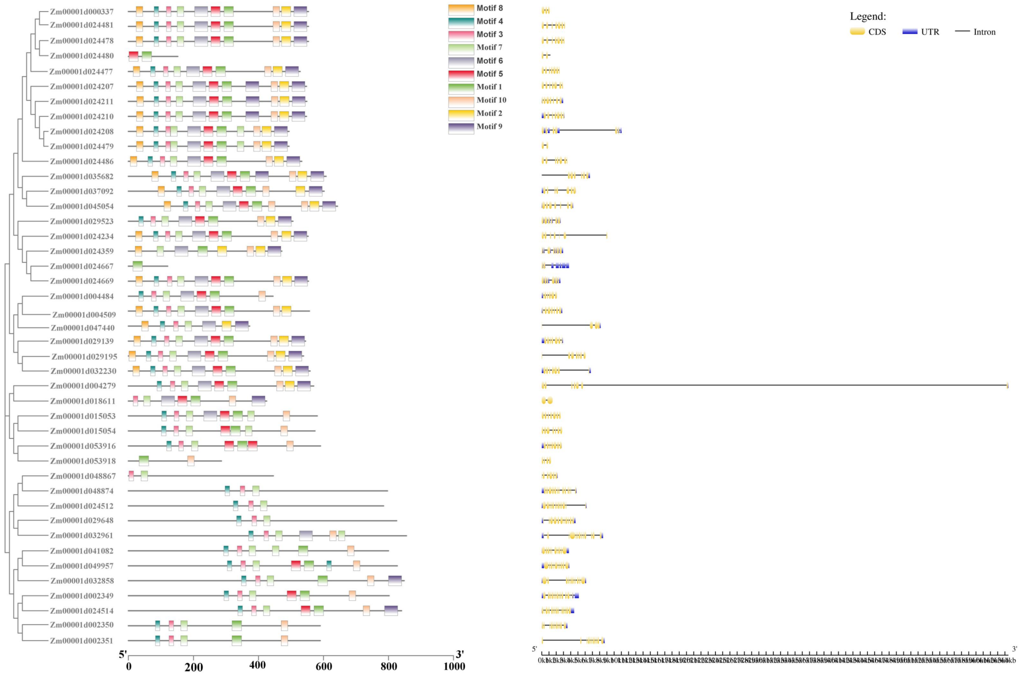

Figure S1 Phylogenetic tree, conserved motif, and gene structure analysis of *TPS* genes. Phylogenetic tree was generated with MEGA-X using the NJ method (Left), 10 conserved motifs analysis was performed using MEME (motif-basedsequence analysis tool) and each motif is represented with a colored box (Middle). The introns-exons organization in *TPS* genes is denoted; yellow boxes represent exons, introns with thin black lines, and blue boxes indicated the untranslated (UTR) region (Right).

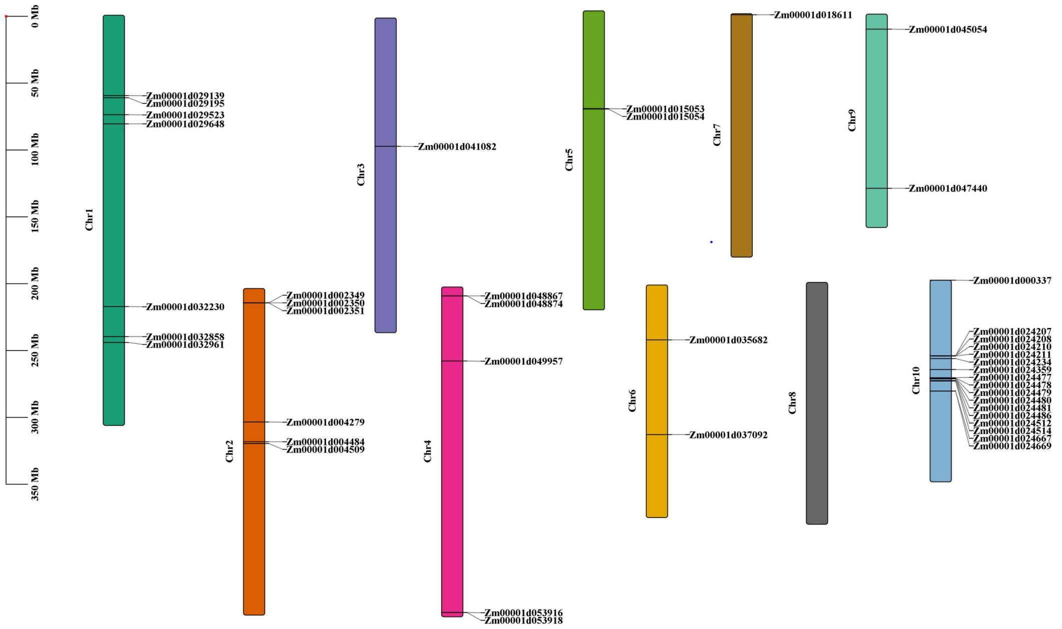

Figure S2 Distribution of the identified 43 *ZmTPS* genes across the maize genome. The ten chromosome groups are shown. All maize chromosomes are drawn to scale based on their actual physical lengths.

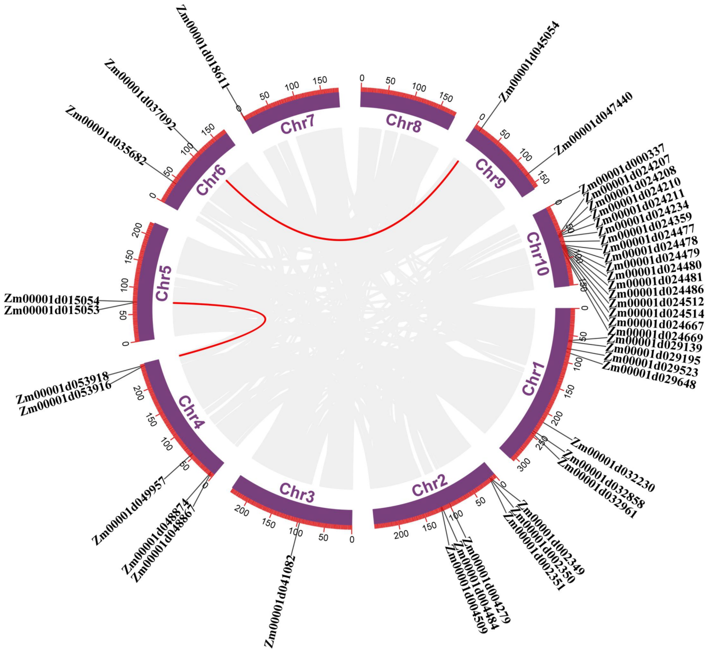

Figure S3 Localization and synteny of the *TPS* genes in the maize genome. The *TPS* genes in maize (*ZmTPSs*) were mapped to different chromosomes. The chromosome number is indicated on the outside. The numbers along the chromosome boxes represent sequence lengths in megabases. Gene pairs with a syntenic relationship are joined by a line.

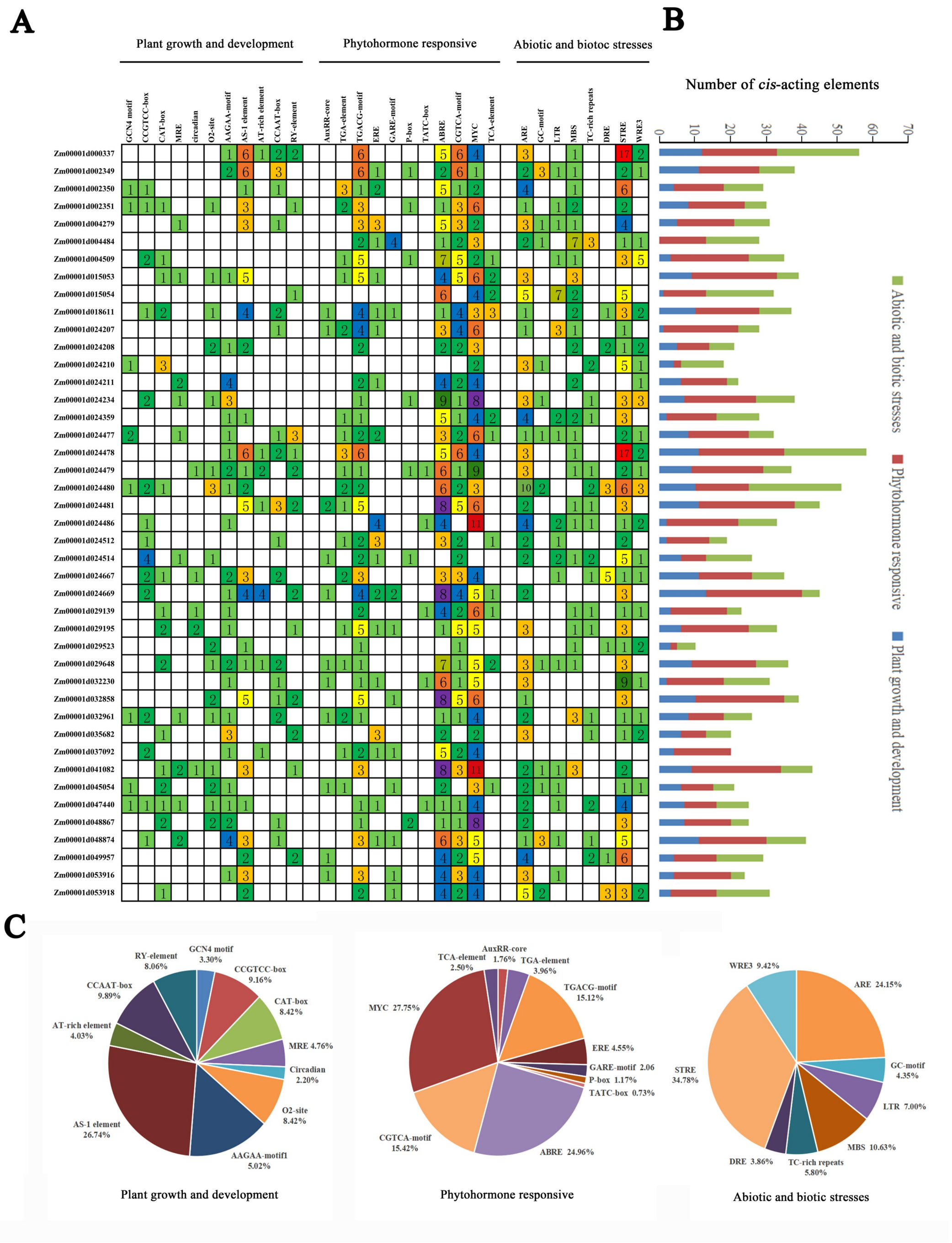

Figure S4 Analysis of *cis*-elements in the promoters of *ZmTPS* genes. (A) Numbers of different *cis*-elements in each *ZmTPS* gene. (B) The sum of *cis*-elements of *ZmTPS* genes in each category. (C) The ratio of each element in different category.

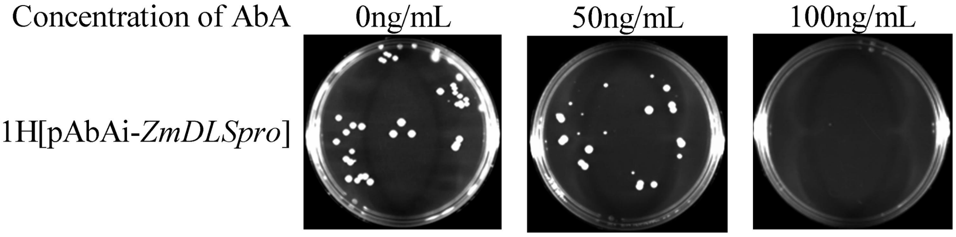

Figure S5 Schematic diagram of pAbAi-*ZmDLSpro* in background level.

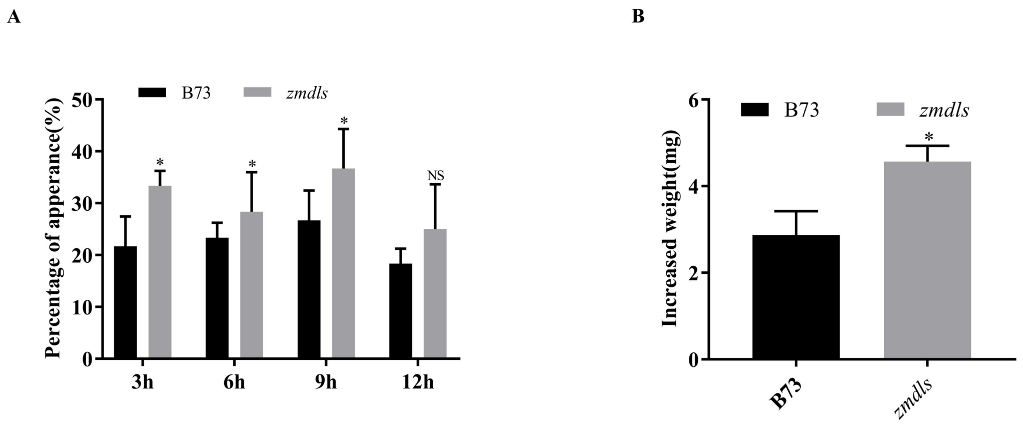

Figure S6 Feeding experiment of corn borer (*O. nabilalis*) on *zmdls* mutant and wild type. (A) Taxis of corn borer (*O. nabilalis*) to *zmdls* mutant and wild type. Bars represent the mean values (±SD) of three biological replicates. Significance level (*$p<0.05$), NS means not significant ($p>0.05$). (B) Weight gain of corn borer larvae when feeding on *zmdls* mutant or wild type. Corn borer weight was measured 4 d after feeding on leaves of *zmdls* mutant or wild type. Bars represent the mean (±SD) of 30 larvae.

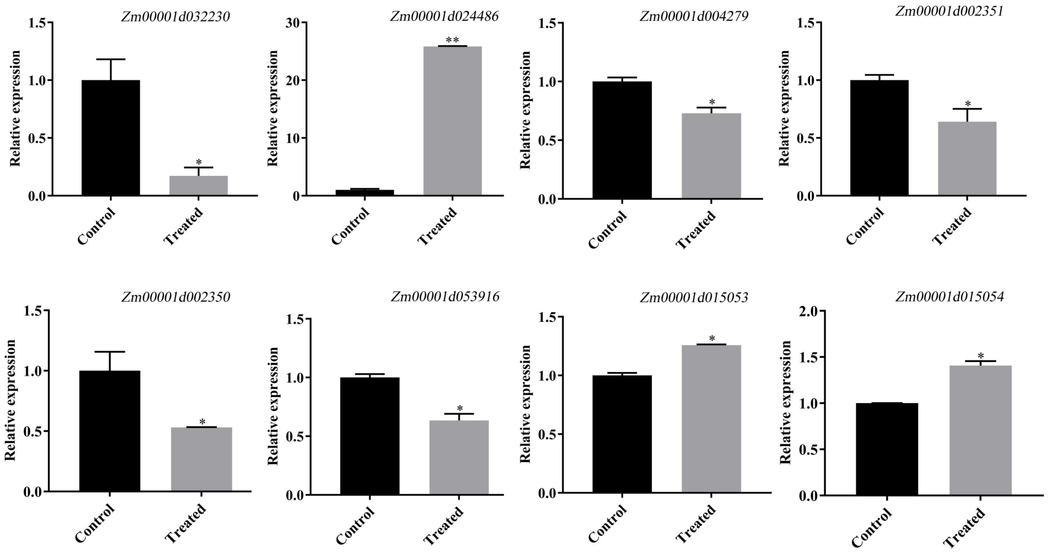

Figure S7 Expression of *ZmTPSs* after maize borer’s bite in the *zmdls* mutant. Bars represent the mean values (±SD) of three biological replicates. * Significant difference at $p < 0.05$, ** Significant difference at $p < 0.01$.

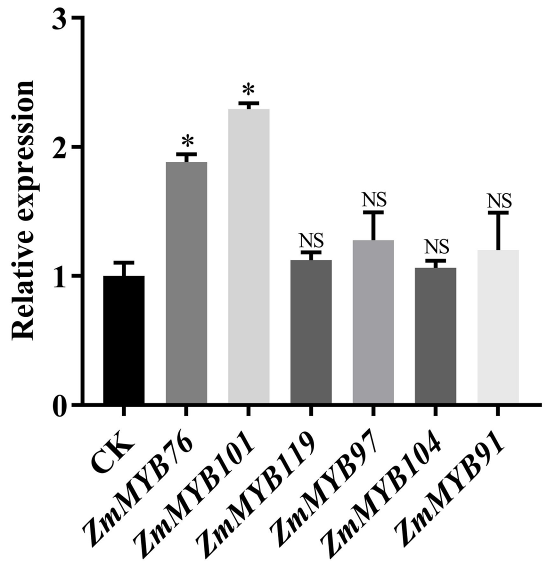

Figure S8 Expression of *ZmMYBs* after maize borer's bite in the wild type. Bars represent the mean values (±SD) of three biological replicates. Significance level (*p<0.05), NS means not significant (p>0.05).

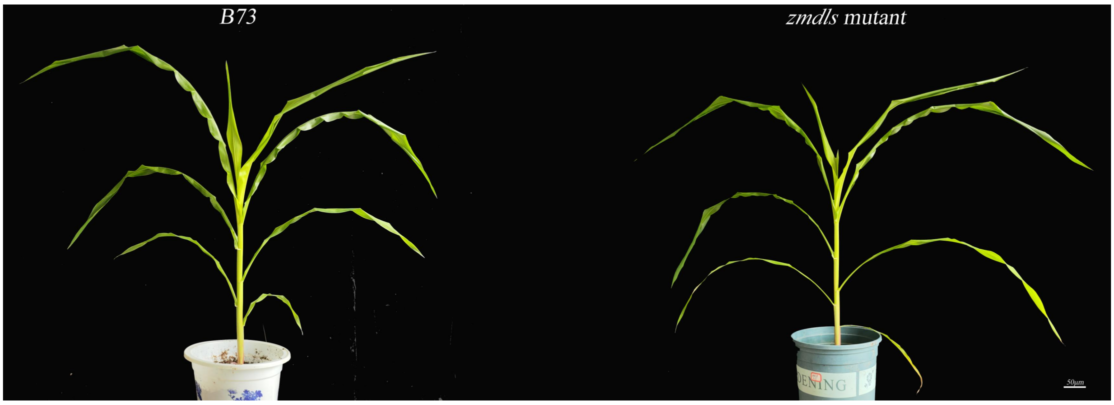

Figure S9 Phenotypes of the maize inbred line B73 and *zmdls* mutant.
