# Supplementary material for: Identification and functional analysis of ZmDLS associated with the response to biotic stress in maize
Source: Front Plant Sci. 2023 Jul 20;14:1162826. doi: 10.3389/fpls.2023.1162826 (PMC10399692; doi:10.3389/fpls.2023.1162826)
Supplement: Supplementary Table 8 — Summary of qRT-PCR on data [file DataSheet_1.zip › Supplementary Table 1.PDF]

Table S1. 43 ZmTPSs identified by bioinformatics analysis and their basic information

Group	Gene ID	Gene name	Length	Molecular weight	Theoretical pI	Genome position	Subcellular localization
TPS-a	Zm00001d000337	None	554	63850.728	4.989	Chr10:66-1163	Chlo
	Zm00001d004484	None	445	51448.939	5.364	Chr2:114598015-114600114	Cyto
	Zm00001d004509	ZmTPS17	557	63294.479	4.921	Chr2:115859006-115861844	Cyto
	Zm00001d024207	ZmTPS6	548	63731.339	5.121	Chr10:56446489-56449417	Chlo
	Zm00001d024208	ZmTPS12	494	57262.916	5.31	Chr10:56531883-56542918	cyto
	Zm00001d024210	ZmTPS11	548	63938.252	4.904	Chr10:56611786-56614909	Cyto
	Zm00001d024211	ZmTPS13	549	63829.202	4.94	Chr10:56725577-56728537	Cyto
	Zm00001d024234	ZmTPS23	553	64436.659	5.229	Chr10:58548223-58557517	Nucl
	Zm00001d024359	ZmTPS22	472	54193.409	6.011	Chr10:66773173-66776124	vacu
	Zm00001d024477	ZmTPS9	528	61314.167	4.781	Chr10:72975155-72977628	Cyto
	Zm00001d024478	ZmTPS4	554	63878.869	5.04	Chr10:73138215-73141345	Chlo
	Zm00001d024479	None	494	57262.916	5.31	Chr10:73296507-73297376	Cyto
	Zm00001d024480	ZmTPS5	152	17987.96	5.18	Chr10:73297790-73298997	Cyto
	Zm00001d024481	None	554	63868.826	4.937	Chr10:73442791-73446010	Chlo
	Zm00001d024486	ZmTPS10	533	61533.515	5.145	Chr10:73709085-73712625	Cyto
	Zm00001d024667	None	121	14267.506	6.896	Chr10:82869683-82873455	Cyto
	Zm00001d024669	None	554	64090.915	5.244	Chr10:82923300-82925885	Chlo
	Zm00001d029139	ZmTPS31	545	61930.873	5.022	Chr1:60187359-60190294	Cyto
	Zm00001d029195	ZmTPS8	539	62025.544	4.85	Chr1:61777169-61783234	Cyto
	Zm00001d029523	None	506	59940.831	5.336	Chr1:74533261-74535868	Cyto
	Zm00001d032230	ZmTPS7	559	63907.653	6.044	Chr1:218044601-218051385	Extr

	Zm00001d035682	ZmTPS15	608	70319.559	6.603	Chr6:40982316-40988980	Chlo
	Zm00001d037092	ZmTPS26	602	67973.118	5.757	Chr6:111878353-111883068	Chlo
	Zm00001d045054	ZmTPS19/STC	643	72109.363	5.788	Chr9:11188093-11192433	Chlo
	Zm00001d047440	ZmTPS21	373	41731.385	4.946	Chr9:130202509-130210660	Cyto
TPS-b	Zm00001d004279	ZmTPS18	570	65725.425	7.465	Chr2:99777895-99842379	Chlo
	Zm00001d018611	None	425	47051.574	6.111	Chr7:845376-846882	cyto
TPS-c	Zm00001d024512	ZmCPS3	785	87641.423	5.645	Chr10:75069682-75075862	Nucl
	Zm00001d029648	AN2/ZmCPS2	825	92761.966	6.027	Chr1:81307314-81312008	Chlo
	Zm00001d032961	AN1/ZmCPS1	855	97565.132	6.368	Chr1:244858884-244867408	Chlo
	Zm00001d048867	None	595	66584.49	5.56	Chr4:6755310-6757503	Chlo
	Zm00001d048874	ZmCPS4	797	90212.944	5.948	Chr4:6825819-6830625	Chlo
TPS-e/f	Zm00001d041082	ZmKSL2	800	89179.11	5.134	Chr3:96077284-96081039	Chlo
	Zm00001d002349	ZmKSL3	802	90809.887	6.39	Chr2:10674212-10679342	Chlo
	Zm00001d002350	ZmKSL5	590	67719.304	6.467	Chr2:10686321-10689881	Chlo
	Zm00001d002351	ZmTPS1	590	67327.759	6.05	Chr2:10715920-10724623	Mito
	Zm00001d024514	ZmKSL6	840	94759.961	5.537	Chr10:75445940-75450421	Chlo
	Zm00001d032858	ZmKSL4	848	95107.217	5.042	Chr1:240465139-240471273	Chlo
	Zm00001d049957	ZmKSL1	827	92385.802	6.832	Chr4:55492468-55496277	Chlo
TPS-g	Zm00001d015053	ZmTPS2	581	65428.444	5.674	Chr5:73220320-73222932	Chlo
	Zm00001d015054	ZmTPS3	574	64779.517	5.675	Chr5:73448400-73451222	Chlo
	Zm00001d053916	ZmTPS2-like	591	67274.819	6.471	Chr4:243626570-243629334	Plas
	Zm00001d053918	ZmTPS2	286	32942.906	4.84	Chr4:243641011-243642284	Chlo
