# Supplementary material for: Identification and functional analysis of ZmDLS associated with the response to biotic stress in maize
Source: Front Plant Sci. 2023 Jul 20;14:1162826. doi: 10.3389/fpls.2023.1162826 (PMC10399692; doi:10.3389/fpls.2023.1162826)
Supplement: Supplementary Table 8 — Summary of qRT-PCR on data [file DataSheet_1.zip › Supplementary Table 2.PDF]

Table S2. Amino acid sequences of 43 ZmTPSs

Gene ID	Amino acid sequence
**Zm00001d000337**	MASHPAHRSSKAADEELPKASSTFHPSLWGSFFLTYQPPTAPQRAN<br>MKERAEVLRERVRKVLKGSTTDQLPETVNLILTLQRLGLGYYYENE<br>IDKLLHQIYSNSDYNVKDLNLVSQRFYLLRKNGYDVPSDVFLSFKTE<br>EGGFACAAADTRSLLSLYNAAYLRKHGEEVLDEAISSTRLRLQDLL<br>GRLLPESPFAKEVSSSLRTPLFRRVGILEARNYIPIYETEATRNEAVLE<br>LAKLNFNLQQLDFCEELKHCSAWWNEMIAKSKLTFVRDRIVEEYF<br>WMNGACYDPPYSLSRIILTKITGLITIIDDMFDTHGTTEDCMKFAEAF<br>GRWDESAIHLLPEYMKDFYILMLETFQSFEDALGPEKSYRVLYLKQ<br>AMERLVELYTKEIKWRDEDYVATMSEHLQVSAESIGANALTCSAYA<br>GMGDMSITKETFEWALSFPQFIRTFGSFVRLSNDVESTKREQTKDHS<br>PSTVHCYMKEHGITMDDACEKIKELIEDSWKDMLEQSLALKGLPKV<br>VPQLVFDFSRTTDNMYRDRDALTSSEALKEMIQLLFVEPIPE
**Zm00001d004484**	MRERAERLKAQVRQAFDAASMDVAGLVTYVDTLERLGIDNHFRDL<br>IGGALDRIRAEELPEHGGGLHIVALRFHLLRQHGIWVSTDVFDAFRD<br>DASGFCSSLCSDDDPRGLLSLYNAAHMAVPGEVVLDDAIAFARGRL<br>LDILRGSKVRSPVSEQITRALDIPLPRFTRRLETMHYIAEYEHEETHD<br>GLLLELARLNFVLVRALHLRELKDLSLWWRDLYNTVKLPYARDRM<br>VEIYFWTCGMLHEEEYSLARMFFAKTFGMVSLMDDTFDVHATLDE<br>CHKLKEAMQRWDESEVYILPEYLRMLYIQTLSNFREFEEILEPNKKY<br>RMAYTKEAYKLCSQNYLKEAIWSSQKYQPSFKEHEELSIMTSGLPM<br>LTILTLMGYGDEATPEAFEWVSSVPEMVRAGSQVTRFLNDLSSYKL<br>GKHKKDMPSSVETYMVENGLTGDLKQVLPL
**Zm00001d004509**	MAPSNIVVQSSSTPPVAGGDEEFAPSVWGDFFVTYATPVSQASEQRM<br>SERAELLKAQVRQAFDAASMDVAGLITYVDTLERLGLDNHFRDLIG<br>AALERIGAEELPEHGGGLHIVALRFRLLRQHGIWVSTDVFDAFREDA<br>GGFCSSLCSDDPRGLLSLYNAAHMAVPGEVVLDDAIAFARGRLLDII<br>SKGEVRSPVSEQITRALDIPLPRFTRRLETMHYIAEYEHEEAHDGLLL<br>ELARLNFVLVRALHLRELKDLSLWWRELYNTVKLPYARDRMVEIYF<br>WTCGMLHEEEYSLARMFFAKTFGMVSLMDDTFDVHATLDECHKL<br>KEAMQRWDESEVSILPEYLRLLYIKTLSNFKEFEEILEPNKKYRMAY<br>TKEAYKLCSKNYLKEAIWSNQKYQPSFKEHEELSIMTSGLPMLTILT<br>LMGFGDEATPEAFEWVSSVPEMVRAGSQVTRFLNDLSSYKLGKNK<br>KDMPGSVETYMVENGLTGDEAAAAIAALLENRWRILNQTRMEIDH<br>TLLPAAQVVLNMARANEIIYLHGRDAYTFGADLKDLVTTLFLKQVL<br>PL
**Zm00001d024207**	MAAPTLTADGPRLGQQEMKKMSPSFHPTLWGDFFLSYEAPTEAQEA<br>QMREKAAVLKEEVRNMIKGSHDVPEIVDLIITLQRLNLDYHYEDEIN<br>EKLTVVYKSNYDGGNLDLVSRRFYLLRKCGYDVSSDVFLKFKDQL<br>GNFVEADTRSLLSLYNAAFLRIHGETVLDEAISFTMRVLQDRLEHLE<br>SPLAEEVSSALDTPLFRRVGTLEMKDYIPIYEKDAKQNKSILEFAKLN<br>FNLLQLRYSSELKECTTWWKELRVESNLSFVRDRIVEVYFWMSGGC<br>YDPQYSHSRIILTKIVAFITILDDTLDSHATSCESMQLAEAIERWDESA

	VSLLPEYMKDFYMYLLKTFSSFENELGPDKSYRVFYLKEAVKELVR
	EYTKEIKWRDEDYVPKTLKEHLKVSLISIGGTLVLCSAFVGMGDVV
	TKKIMKWVMSDAELVKSFGIFVRLSNDIVSTKREQREKHCVSTVQC
	YMKQHEITMDEACEQIKELTEDSWKFMIEQGLALKEYPIIVPRTVLE
	FARTVDYMYKEADKYTVSHTIKDMLTSLYVKPVLM
**Zm00001d024208**	MAAQTLTADGPRLGQQEMKKMSPSFHPTLWGDFFLSYEAPTEAQE
	AEMRERAGVLREKVRSMIKGSHDVPEIVDLIITLQRLNLDYHYEDEI
	NEKLTVVYNSNYDGGNLVLVSRRFYLLRKCGYHVSSDTRTLLSLYN
	AAYLRIHGETMLDEAISFTTRCLQDRLEHLESPIAEEVSSALDTPLFR
	RVGTLEMKDYIPIYEKDAKQNKSILEFAKLNFNLLQLLYSSELKECTA
	WWKELCVESNLSFVRDRIVEVYFWMSGGCYDPQYSHSRIILTKIVAF
	ITILDDTLDSLANSYESMQLAEAVERIHEGFLHVKELVREYTKEIKW
	RDEDYVPKTLKEHLKVSLISIGGTLVLCSAFVGMGDVVTKKIMEWV
	MSDAELVKSFGIFVRLSNDIVSTKREQREKHCVSTVQCYMKQHELT
	MDEACEQIKELTEDSWKFMIEQGLALKEYPIIVPRTVLEFARTVDYM
	YKEADKYTVSHTIKDMLTSLYVKPVLM
**Zm00001d024210**	MAAPTLTTDGPRLGQQEMKKMSPSFHPTLWGDFFLSYEAPTEAQEA
	EMRQRAEVLREEVRNMIKGSHDVPEIVDLIITLQRLNLDYHYEDEIN
	EKLAVVYNSNYDGGNLDLVSRRFYLLRKCGYHVSSDVFLNFKDQY
	GNFIEVDTRSLLSLYNAAYLRIHGETVLDEAISFTTRCLQDRLEHLES
	PIAEEVSSALDTPLFRRVGTLEMKDYIPIYEKDAKQNKSILEFAKLNF
	NLLQLLYSSELKECTTWWKELRVESNLSFVRDRIVEVYFWMSGGCY
	DPQYSHSRIILTKIVAFITILDDTLDSHANSYESMQLAEAVERWDESA
	VSLLPEYMKDFYMYLLKTFSSFENELGPDKSYRVFYLKEAVKELVR
	EYTKEIKWRDEDYVPKTLKEHLKVSLISIGGTLVLCSAFVGMGDVV
	TKKIMEWVMSDAELVKSFGIFVRLSNDIVSTKREQREKHCVSTVQC
	YMKQHELTMDEACEQIKELTEDSWKFMIEQGLALKEYPIIVPRTVLE
	FARTVDYMYKEADKYTVSHTIKDMLTSLYVKPVLM
**Zm00001d024211**	MAALTQTTDGPRLGEQEMKKMSPASFHPTLWGDFFLSYEAPTETQE
	AQMREKAGVLKEEVRNMIKGSHDMPEIVDLIITLQRLNLDYHYEDE
	ITEKLTVVYNSNYDGGNLDLVSRQFYLLRKCGYHVSSDVFLNFKDQ
	YGNFIGADTRSLLSLYNAAYLRIHGETVLDEAISFTTRCLQDRLEHLE
	SPIAEEVSSALDTPLFRRVGTLEIKDYIPIYEKDAKQNKSILEFAKLNF
	NLLQLLYSSELKECTSWWKELRVESNLSFVRDRIVEVYFWMSGGCY
	DPQYSHSRIILTKIVAFITILDDTLDSHANSYESMQLAKAVERWDETA
	VSLLPEYMKDFYMYLLKTFSSFENELGPDKSYRVFYLKEAVKELVR
	EYTKEIKWRDEDYVPKTLKEHLKVSLISIGGTLVLCSAFVGMGDVV
	TKKIMEWVMSDAELVKSFGIFVQLSNDIVSTKREQREKHCVSTVQC
	YMKQHELTMDEACEQIKELIEDSWKFMIEQGLALKEYPIIVPRTVLE
	FARTVDYMYKEADKYTVSHTIKDMLTSLYVKPVLM
**Zm00001d024234**	MAADEARSVSRLHSEEDMHGKHHSTLWGDFFLHHVPCRPGQYLIM
	KDNVEIMKEEVKKMLLDVGSSDLSHKLDCIDTLERLGLDYHYTKEI
	DELMCNVFEARDQDLDLTTTSQLFYLLRKHGYHISSDVFLKFRDDK
	GDIVTNDARCLLRMYEAAHVRVNGEEILDNILIHTKRQLQCIVDDLE

	PTLQEEVRYALETPLFRRLNRVQARQFISTYEKSTTRINMLLEFSKLD
	FNILLTLYCEELKDLTLWWKEFQAQANTTIYARDRMVEMHFWMMG
	VFFEPQYSYSRKMLTQLFMIVSVLDDLYDSHCTTEEGNAFTAALQR
	WDEEGVEQCPTYLRTLYTNIRATIKAIEEDLNFQNNKHAKLVKGLII
	DMAMCYNAETEWRDKKYVPATVDEHLKISARSSGCMHLVSQGFIS
	MGDVATSEALEWASTYPKIVRAVCIIARLANDIMSYKNLFYLQREAS
	NNTMVSTVQTCAKEYGTTTVEQAIEKIRELIEEAWMDITHECLRQP
	QPKALLERAVNLARTMDFLYKDADGYTDSRSIKGILDSLYVHLID
**Zm00001d024359**	MATSGPVLLVSQAQQRKSRTYTPSPWGDFFLHHVPCTPSQLLSMKE
	RAKVKEQQVRQIIHETIASSDLLHVFEKFRDESGAIVSGDDASCLLM
	LHDAAHARTHGEEILDGIVAWSKSRLQSLVVQKNLLEPELAEEVRC
	ALETPRFRRVKRVEARRYIPVYEKKSTRDETILEFAKLDYNISQAIYC
	DELKELTIMVELHFWMLGVIHDPYCSRIMIVKIFVFISLFDDLMDNY
	STTEESNVFTAAMERWDEQMAQQLPAGLKALLVRIFNATNKIEEEL
	KLQGNMHAELIKKMVINSAKFYHAEVKWRDEHFVPTSLEEHLQKSI
	PTSACMQMANLALVSLGNVTTREDVEWVFTFPKIIRGACIVGRIAN
	DIVSHEREQASSDHVASTVQTCMKQYNVTIKEANEKLRAVIEEAWM
	DIVQDSLEQERPRALLDIAINVARVADFMYKREDAYTLSFVIKDIIAS
	MYIYSV
**Zm00001d024477**	MAPPPPALAEETAAFHPSLWGDFFLTYQPPTAPQQACMKERAEVLR
	EDVRKILMGPCELPETLGLILTLQRLALDYYYENEIDKLLHCIYDDD
	DSDYDDKDLNLVSLRFYLLRKNGYDVSSDVFLVFKTEEGSFAYADD
	TASLLSLYNAAYLRKHGEEVLDEAISFARRRCLEAGILRTPLFRRVGI
	LEARNYIPIYEKEAARNDAVLELAKLNFNLQQIAFCEELKHCTLWW
	KDFLAKSKMAFVRDRIVEVYFWMNGACYDPPYSRSRIILTKITSLVT
	IIDDMFDTYGTTEDCTKFHEAIGRWDESALPLLPQYMKGFYLFQLET
	FRSFEDELGPENSYRVHYLKKSMERLVQQYYREIKWRDENYVPKT
	MTEHLQVSMESIGSVALACAAYVGMGDVITKETLDWVLSYPQFLTT
	YGIVRLSNDVVSTKREQTKDHSPSTVHCYMKEHGTTMDDACEKIK
	ELIEDSWKDMLQQSLALKGLPKAVPRTVFDFLRTADNMYKNCDAF
	TSSEVLREMIRLLFVEPIPE
**Zm00001d024478**	MASPPAHRSSKAADEELPKASSTFHPSLWGSFFLTYQPPTAPQRANM
	KERAEVLRERVRKVLKGSTTDQLPETVNLILTLQRLGLGYYYENEID
	KLLHQIYSNSDYNVKDLNLVSQRFYLLRKNGYDVPSDVFLSFKTEE
	GGFACAAADTRSLLSLYNAAYLRKHGEEVLDEAISSTRLRLQDLLG
	RLLPESPFAKEVSSSLRTPLFRRVGILEARNYIPIYETEATRNEAVLEL
	AKLNFNLQQLDFCEELKHCSAWWNEMIAKSKLTFVRDRIVEEYFW
	MNGACYDPPYSLSRIILTKITGLITIIDDMFDTHGTTEDCMKFAEAFG
	RWDESAIHLLPEYMKDFYILMLETFQSFEDALGPEKSYRVLYLKQA
	MERLVELYSKEIKWRDDDYVPTMSEHLQVSAETIATIALTCSAYAGM
	GDMSIRKETFEWALSFPQFIRTFGSFVRLSNDVVSTKREQTKDHSPST
	VHCYMKEHGTTMDDACEKIKELIEDSWKDMLEQSLALKGLPKVVP
	QLVFDFSRTTDNMYRDRDALTSSEALKEMIQLLFVEPIPE
**Zm00001d024479**	MAAQTLTADGPRLGQQEMKKMSPSFHPTLWGDFFLSYEAPTEAQE

	AEMRERAGVLREKVRSMIKGSHDVPEIVDLIITLQRLNLDYHYEDEI
	NEKLTVVYNSNYDGGNLVLVSRRFYLLRKCGYHVSSDTRTLLSLYN
	AAYLRIHGETMLDEAISFTTRCLQDRLEHLESPIAEEVSSALDTPLFR
	RVGTLEMKDYIPIYEKDAKQNKSILEFAKLNFNLLQLLYSSELKECTA
	WWKELCVESNLSFVRDRIVEVYFWMSGGCYDPQYSHSRIILTKIVAF
	ITILDDTLDSLANSYESMQLAEAVERIHEGFLHVKELVREYTKEIKW
	RDEDYVPKTLKEHLKVSLISIGGTLVLCSAFVGMGDVVTKKIMEWV
	MSDAELVKSFGIFVRLSNDIVSTKREQREKHCVSTVQCYMKQHELT
	MDEACEQIKELTEDSWKFMIEQGLALKEYPIIVPRTVLEFARTVDYM
	YKEADKYTVSHTIKDMLTSLYVKPVLM
**Zm00001d024480**	MERRWWNEMIAKSKLTFVRDRIVEEYFWMNGACYDPPYSLSRIILT
	KITGLITIIDDMFDTHGTTEDCMKFAEAFGRWDESAIHLLPEYMKDF
	YILMLETFQSFEDALGPEKSYRVLYLKQAASILHIIYPLMSALYVHESI
	LKEHTVIVII
**Zm00001d024481**	MASPPAHRSSKAADEELPKVSSTFHPSLWGSFFLTYQPPTAPQRANM
	KERAEVLRERVRKVLKGSTTDQLPETVNLILTLQRLGLGYYYENEID
	KLLHQIYSNSDYNVKDLNLVSQRFYLLRKNGYDVPSDVFLSFKTEE
	GGFACAAADTRSLLSLYNAAYLWKHGEEVLDEAISSTRLRLQDLLG
	RLLPESPFAKEVSSSLRTPLFRRVGILEARNYIPIYETEATRNEAVLEL
	AKLNFNLQQLDFCEELKHCSAWWNEMIAKSKLTFVRDRIVEEYFW
	MNGACYDPPYSLSRIILTKITGLITIIDDMFDTHGTTEDCMKFAEAFG
	RWDESAIHLLPEYMKDFYILMLETFQSFEDALGPEKSYRVLYLKQA
	MERLVELYTKEIKWRDEDYVATMSEHLKVSAESIGANALTCSAYAG
	MGDMSITKETFEWALSFPQFIRTFGSFVRLSNDVESTKREQTKDHSP
	STVHCYMKEHGITMDDACEKIKELIEDSWKDMLEQSLALKGLPKV
	VPQLVFDFSRTTDNMYRDRDALTSSEALKEMIQLLFVEPIPE
**Zm00001d024486**	MDATAFHPSLWGDFFVKYKPPTAPKRGHMTERAELLKEEVRKTLK
	AAANQITNALDLIITLQRLGLDHHYENEISELLRFVYSSSDYDDKDL
	YVVSLRFYLLRKHGHCVSSDVFTSFKDEEGNFVVDDTKCLLSLYNA
	AYVRTHGEKVLDEAITFTRRQLEASLLDPLEPALADEVHLTLQTPLF
	RRLRILEAINYIPIYGKEAGRNEAILELAKLNFNLAQLIYCEELKEVTL
	WWKQLNVETNLSFIRDRIVECHFWMTGACCEPQYSLSRVIATKMTA
	LITVLDDMMDTYSTTEEAMLLAEAIYRWEENAAELLPRYMKDFYL
	YLLKTIDSCGDELGPNRSFRTFYLKEMLKVLVRGSSQEIKWRNENY
	VPKTISEHLEHSGPTVGAFQVACSSFVGMGDSITKESFEWLLTYPEL
	AKSLMNISRLLNDTASTKREQNAGQHVSTVQCYMLKHGTTMDEAC
	EKIKELTEDSWKDMMELYLTPTEHPKLIAQTIVDFARTADYMYKETD
	GFTFSHTIKDMIAKLFVDPISLF
**Zm00001d024667**	MIEAIRAALGSTGDGEINISAYDTAWVALVKSLNGDEGPQFPSCIDWI
	AQNQLPDGSWGDGIFFLVQDRIISTLACIIALKSWKIHDDACKKGTL
	STALSNNNKPSYVLYRATYIYVTHEEYILLNRHWSEEGLAFTKGCRV
	KDIDDTAMGFRLLRLHGYHVSPCVFKRFENGDGQFVCYARQSNQS
	VSAMYNLYRAADQGSFPGDDPVLQRAKSYSRAFLRERRASDQLND
	KWIISEGLPSEVGYGLDFPWEACLPRIETRMYLEQYGGSHDVWIGK

	VLYRMNMVSNDMYLKAAKADFGNFQRLCRLEWHDLKRWCDKIS NIETYGVASRGALLRAYFLAAACIFEPSRAAERLAWARAAVLAEAIS CCLLSNVDDACACNKTTAEWLVQEFTNGDDIADSYEYSPARRDDD GPNSPAWGASSLAGVLRELVNLQASGNAAVAGCLRGAWIEWLMAW TGKETEKASRAGDTALLLARTVEICSGRRCGTEKDLDLLLRDYSKL QQLTRCICSRLATEAPALNGETMDKIDALDRMVGLEMRELAQCVFR SGGSSVDRETRQTFLHVTKSYYYVALCSPETVEHHISKVLFEDVV
**Zm00001d024669**	MTTSVPVPLASQPQAPQRKPRTYTPSPWGDFFLHHTPCTPSQLLSM KERAKVKEQQVRQIIQETMASSNLLRKLELVDTLQRLGVDYRYYEE IDQLLRSVYDDGDKDGGGSDDLYLTSLRFYLLRKHGYTTSSDVFEK FRDERGSVSSDDASCLLMLYDAAHTRTHGEEILDDIIAWSKSRLQSL LEEKNLEPELAEEVRCALETPRFRRVKRVEARRYISAYEKKGGRDGT ILEFAKLDYNISQAIYCDELKELTVWWKDFQSHIDMRFTRDRLVELH FWMLGVIHEPYYSYSRIMMTKFFIFGNFFDDLYDNYGTTEESDVFTA AMERWDEQIAHQLSAGLKVLLASILNATNKIEEELKLQGNMHAELV KKMVIDTAKSYHAEVKWRDEHFVPATVEDHLQISIPSSGCMQITNLA LISSRGNMTTREDVEWVFTFPKIVRGACIVGRICNDIMSHEREQASL DHVASTVQTCMKQYGVTTEEANEKLRAVIEMAWMDIVQDNLEQER PIALLDTMINVARIADFIYKREDAYTLSFSLKGIIGSMYVYSV
**Zm00001d029139**	MASHNASVSVPEMASGFHPTVWGDFFIDYSPQPSQKSEEWTAERMS QLKEKIAGLFGACTTTAEQLNLVDTLQHLSIDHHFSTQILSVLTSAHA AEELNSGGACLHDVALRFRLLRQQGFWVSPEVFDRFRDENGSFDVG VADDDARGLLSLYNAAYLLTHGEAELEEAALFARQRLESMSMRGSL EYPLAQQVSRALHLPLPRTVRRVETLHYVSEYGGDPTHEHEHDPSV LEFARLDFELLQRLHVKELKALSRWWKDLYNEAGVTYSRDRVVEC YLWSYTAYYEEEHARARMILAKMIALIILTDDTYDVRATLEECRRW DESAVSLLPDYLKKFYLKLMNIFEEFEDELEPHEKCRVAFSRKAFQT LSSNYLQEAEWFHGRYKPRFEDQVKVSTVRSGAPFAAVGLLVGMG GDVATQEALEWAMGCTDAVKAFAEVTRFMNDLASYKRGKNKNDV DSSVECYISEHGVTAEVAFAQISSLIEDAWKTINGARFENSKLLPAVQ RVADITASMPLMYGDNKDAFTFSDGLKGLIERLFLNRP
**Zm00001d029195**	MAPKTVWGYFFIDYIPEPLQVSDKQRVVELKGEVARLFEDCNCKDV VERMNLVDVVQRLGIDHHFKEQIDTALKNIQGAEFNSSDLHEVSLRF RLLRQHGLWVPADQFDKFRRQEDGSFSSDIADDPKGLLGLYNAASL LIHGEEVLEEALLFARRHLESIRRGGGLHDSPYLSEQVGRSLKIPLPR TLKRLEAVSYIPEYSSADDTTYIHPEILELARLDFNLLQHVHQNELRT VTQWWKGLCDVIGPDYGRDRIVECYFWAFSMYYEEEHARARMILA RLIMLASLLDDTFDDRATLQECRELNKAIERWDESDDISLLPECIQKF FLEVIRNFAEFEDELEAHEKYRVAYARKAYQLLSKSYLQEVEWCHQ GYTPSFDDHVSVSTASAGIQVLCVGMLVGMGDAATKEVFEWMIGS NNRVVRACAEVTRFMDDMADFKRGKNKTDVATTVECYMKEQNVT GEVAFDKIGSFVEDAWKTLNQAALVGDRALLPVVQRVAGLAMSMM VFFHGKIDRYTDSEHLKETLEDLFVNHVPLC
**Zm00001d029523**	MKERRDVLIKEAKKRLTGKHDDPLAEMNLIDAVQRLGVSYHLQDEI

	HASLQKLNSMEFSNESFHEISLQFWLLRQERYCMSCDVFQSFMDDQ<br>RKLNANIKSDVRALLALYEAAHLGTPTEQFLTEAQRQTTSLLRSMV<br>GHLEKPLADKVRHALRTPSFRRMKRLEARLYIPLYEEDKEDCDEMIL<br>ELAKLDFYLLQRIHREEVKEICEWYHGLDSPRKLFYARHRPTEAYF<br>WALGVYYEPQYAKARKLLAKFIATITPYDDTFDNYGIWEELQPFAD<br>VMQRWDMKEAEGLNECYSDFARFMFGTMIEIENALPKDIGRRNVN<br>FIRDIINEVCKGYVTEIGWRDTKYIPPLEEHLKVTLITCFYWGINCTA<br>FVVFEENVTEEILKWMSRFPQIVKDSCIISRLMDDIVAHEFETERDNV<br>ATAVTCYMKQYKTTKEEASDALWNSVEQAWKSMNHEYLTWTSIPS<br>SLLIRVINLARMMETMYKEIDGYTDSKLLKEWISMLLDQPIPF
**Zm00001d032230**	MATTGTTSMPAPVFHPTVWGDYFIKFVPEPLQVSDETMAERIRHLR<br>EEVSGMFQACKNVVDKTNLVDVVQRLGIDHHFEEQIATALASIHSA<br>GLFNSSSLHEAALRFRLLRQQGFWVPADELVKFIKNEDGSFIDGITN<br>DPKGLLSLYNAAHLVTHDEGTTTLEDAIAFARQHLEAARRCSLKSPL<br>AEQVGRALGIPLPRTLKREEAIAFIPEYSSQQDQQVYSPVILELAKLD<br>FNLLQHLHQEELKEISHTYVRTYVLNGRWWKDLSGEIGLGYVRDRI<br>VECYFWSYTVHYERGQARARMILAKVFMLTSLLDDTYDVHATLEE<br>ARELNKAIQRWDESDVSLLPEYLKKFFVKVISNFREFEDELESHEKY<br>RNVYNIKGFQTLSKHYLQEAEWFHHGCTPSFKDQVNVSVITGGAQ<br>VLSIGLLVGMGHEATREAFEWAIGDTDAIWACGEVSRFMDDMSAFK<br>NGRNKMDVASSVECYIKEHNVPSEVALARINSLVEDAWKTINQAPF<br>KYPALFPVVQRVTSLAKSMTLLFLDKRDAYTYSKDFQTTLETHFVR<br>HIPL
**Zm00001d035682**	MASFSLKPQHNGSSSRIYHTICSATASEWRSLLRSRSSVSWSWKKHT<br>RLRLRCCQGSNGGDDSRLSQNTGIFHPSIWGDFFLGCSSSPEEAAAS<br>SQQKAWMERCDELKEEVGRLMVVEANSPLHERLHFIDALESLCLD<br>HLLKEEIGAALTQIETAGVSDDCDIGTVSLWFYLLRKHRRRASPDVF<br>VRFRDAEGGFQANDPGDLLRLYNAAHFRTHGETILDEAIAFARSRLE<br>MMLPYVKGPLLAHEVRSSLEIPLPRRVRIYESKYYIHAYETSGTLHE<br>KVLQLAKLNSNVLQLHHQQELKILTRWWEDMQIGSKLPFARDRIVE<br>CFLWILGVYYEPCHSRARIILTMVIAIVTLLDDIFDSFATPQECEILTNC<br>IESWDPKGAQDLPGCMKFALEKILDSYQTITNMLHREDKYRMTYLR<br>HFTEDLVRSFNMEVKMLQKGYIPDSVEEHLKVSLRTGGCPILSCACF<br>VGMDDIATKDFFDWISSVPKMVRALSIILRLADDLHSYEREQLIPHV<br>ASTIDTYMKKHNVSIEVAQENIHKLKEESWKDLNSEWLNPCNDVYP<br>KQLLERIFNLARTMEFMYNQEDNFTNCHNLKDTIRCLLVEPYRIHI
**Zm00001d037092**	MAGITGVMNMKLAARPSSGRHSRGCRPAVVPSAGKQMLLVRRHPP<br>GSASWPTRATGGGGGGVPAGATAADSSGQAKEEEEEDRASRNTSSF<br>EPSIWGDFFLTYSSPLATSSAQKARMVHRAEQLKKQVAKLIAASGA<br>CSLYHRIHLVDALERLCLDYLFEDEINDMVTQIHNVDVSGCDLQTV<br>AMWFYLLRNHGYRVSSDVVFAKFRDEQGGFAANNPRDLLNLYNA<br>ACLRTHGETILDEAASFTSKCLKSLAPYTYMEASLASEIKRALEIPLP<br>RSVRIYGAKSRIAEYGNQTEANELVLELAKLNYNLVQLQHQEELKII<br>TRWWNDLELQTRLSFARDRVVECYFWMVGVYFEPSYSRARVILSK

	VLAIVSLLDDTYDVYGTSQECELFTKCIESWDPAATGGRLPGNMKFI<br>FAKILDTCQSFEDELAPDEKYRMHYLKTFIIDLVRAYNEEVKWREQ<br>GYVPATVEEHLQVSARSGGCHLLSCTSFVGMGDVADQEAFEWREK<br>MSSHVASTMESCMKEHQVPLEVARVKIQETIDETWKDFNEEWLNLN<br>TNSHLPRELLERIFNLTRTMVYIYQQDDAYTNCHVIKDTINSLFVEPV<br>SIT
**Zm00001d045054**	MVAAASTTQAHWQMTGSSYACTCTAFRSISTKLAARSSAGRRSRGR<br>RSAVDPSAGGPPLPPPPVVIAGAVASPGKRMPPSPIGCTSRAATASSSR<br>QAKEDDGASRNVSGFEPSIWGDFFLTYSSPLATSSLHKAWMVHRAE<br>QLKEEVAAKLVAASSACSLHRRIHLIHVLERLCLDHLFEDEISDMLA<br>RMNDVDVSGCDLQTVAMWFYLLRKHGYSVSSDVFAEFRDEQGGG<br>FTANSARDLLSLHCAACLATRGETILDEAISFTAKSLKSTAPYTGAAS<br>LACEIKRALEIPLPRRVRIYEAKSRIAEYGREAEADELVMELAKLNY<br>NLVQLQYQQELKIITRWWNGLELQSRLSFARDRVVECYFWIVGVYF<br>EPSYSRARIILTKVIAIVSLLDDTYDVYGTPQECELFTECIESWDPAVA<br>GGLPENMKFIFGKILDTYQSIEEELAPEEKYRMPYIKNFIIDLVRAYN<br>KEVRWREQGYVPATVEEHLQVSARSGACHLLSCASFVGMGDVAGE<br>EAFEWVCSVPKLVQALCIILRLSDDLKSYEREKMAPHVASTIESCMR<br>EHEVPLEVARVKIQEMIDETWKDFNEEWLNMNKHQPAELLERIFNL<br>TRTMVYMYKNDDAYTNCHVIKDTINSLFVEPVSIA
**Zm00001d047440**	MQQQLPTVCDAKHGWCPLPRVNQQSKADGTAVAAAACHGAFEPS<br>VWGDFFVTYTSPSFSQEESEEQMGERANFLKGEVRKKFEAAAMSAI<br>DAAMLVDAVVHLGIDHCFREEIATALRSVHEDEEGEFGSCDDLHTVA<br>VRFLVLRQHGLWVSADVFDKFRDDKGSFSKSLLCSNPRGLLSLYNA<br>AHMAVTPEEKVLDDAIAFARSHLVEAMIGELRSPMVEQVSRSFDIPL<br>PRFSRRLESMHYIAEYGQEEEGHDAQILELARLEFELVRSLHLRELR<br>EICRNKKDVANAHECEYGATGEEAFAFIANMTENAWRKINQACME<br>MDPAMLPAFKVAVVDLSRSIEIIYLGGKRDAYTFGSNLKDLVTSLFLK<br>PCA
**Zm00001d004279**	MSLRTTAWLSARPGHHQPVQCLSSPEKPRRRSANYQPTSWDYDALL<br>SLDSGRPGDQVHPSSYYKMKRRIRDMLLKKVEPSSKLQTIDAMQRL<br>GISYHFEDEISNILYSISNESTKAQYTYDNIAFTALKFRLLRENGFPTIL<br>GPLGYHNYEKFITKTPSLEDANTLLLLYEASYLAFGDEEKLDVARTF<br>SAKALKQLMPFMLPHLREGVAHSLELPLHWRASRLETRWFIDYYAR<br>DRNMCPLLLQFAELDFNQVQYEHQKDLAVVTRWWKKIGLGEKLPF<br>ARDRLMECFHYANGIVWDPKLGTCRQMLAKVSNLIVYLDDLYDVY<br>GTIEELVLFTNAIARWDAIPNETLPEYMKALYSIIYDTSNEVAEHALK<br>KHGCSMHYLLQKSWYDICLAFLVEAKWHHGNYRPSIQKYLENGW<br>VSSSAPLLLLHAFAMLDSVVNMSTISPLQTKLRLVQHVSLILRLCND<br>SATHLDELGRGDAPSFIALHMIENGGNENDSRKAMQEHILKSWKVI<br>NEEAFDSCQYSMPFNKACIDLARISHCVYQGGDGFGTPNGLKKKQI<br>RDLFLDPIDICNKQ
**Zm00001d018611**	MQQQQPLDLHSAALLFRMLRGLGIPASTDMLMSALLREESGKLAA<br>DSDGLLALYQASYLAFPGETELDQARAFAVAKLAGRRDDGSADSCP

	SSTHLSLPLHWTAPRLQAMWSLKDQVERGRPDHQDQAIILQLAQLD<br>FNLVQALHRRELAEVTRWWKETSRLGEHAFARDRVVECFFCAACIA<br>PEPRLAECREVLAKAGALIVHLDDIYDVYGTPDELRAFTDAVASWE<br>HSATRTTHSPRRSWWRRSGSSRDSGHMPSFAQYVSNGWVTSTGPLL<br>LLHALPAAAAIPRPPIITTAACTVDYYPRLVELSSTIFRLCNDCASHEA<br>ESERGDAPSSIACCMAEPWCAGGEEQARAAVQGVIAETWKLLNRE<br>VCCSCAEHEESLTTMANLCLNLARVIHCIYQDGDGITTPTHRMKRM<br>VKDLLFNPIL
**Zm00001d024512**	MDLPRPQAPCCRRPADLRPAPCCIDAPRPSDLRGCAPAAGALWKPS<br>ASYPRAAVPPASVRLHDISVRGEEDEQKEMLQQMTDGVRAMLGSIG<br>DGAINISAYDTAWVALVKSLEGGNGPQFPSSLRWIVENQLPDGSWG<br>DEEFFLVYDRMINTLACVIALESWEIHADMCEKGLSFIRENLWRLEH<br>SGSDDWMVVSFEITFPQLLEMARDLGLDVPCDEPSLRAIYARRDAK<br>LARIPKELLHASPTTLLLSIEGMPGLDWERLLKLQCSDGSFMSSPAPT<br>AYALMQTGDTKCLEFLNGIVSKFSGGVPFTYPVDLFEHLWVVDRIE<br>RLGIGRHFTGEIKECLEYVHRYWGDEGLPATRDGPVSDVDDTAMGF<br>RLLRLHGYDVSPSVFKHFEQDGAFYCYPGQSNKSVTAMYNLYRAS<br>QVAFPGEDELRRAEAYSREFLCRRRASGELKDKWVIPKDLPGEVAY<br>ALDVPWKASLPRIETRMYLEQYGGADDVWIGKVLYRMSLVNNELL<br>LRTAQADFRSFQRQCKLEWHGLRKWASRRNLQAYGVTSNSTLRSY<br>FLAAASIFEPDRATERLGWARTAVLAEAVSSCLRDGRCSRADGMLRE<br>LTSGIHLRNDDNPAASLVHALHELIGLLAFDNASYNSLLDAWKQWL<br>AMWTAQGHEGSVALLLVRTVEICSGRRRSASATDDGQAHSLSEYSQ<br>LEQLTSSVCSKLAAAGTGDGDDLSPASVEDAADRHRVDLEMQTLA<br>RCVLRSRSSIDAVTRQTFLHVARSFYYVAHCSARTVDAHISKVLFED<br>VV
**Zm00001d029648**	MVLSSSCTTVPHLSSLAVVQLGPWSSRIKKKTDAVAVPAAAGRWRA<br>RARAQDTSESAAVAKGSSLTPIVRTDAESRRTRWPTDDDDAEPLVDE<br>IRAMLTSMSDGDISVSAYDTAWVGLVPRLDGGEGPQFPAAVRWIRN<br>NQLPDGSWGDAALFSAYDRLINTLACVVTLTRWSLEPEMRGRGLSF<br>LGRNMWKLATEDEESMPIGFELAFPSLIELAKSLGVHDFPYDHQAL<br>QAIYSSREIKVKRIPKEVMHTVPTSILHSLEGMPGLDWARLLKLQSS<br>DGSFLFSPAATAYALMNTGDDRCFSYIDRTVKKFNGGVPNVYPVDL<br>FEHIWAVDRLERLGISRYFQKEIEQCMDYVNRHWTEDGICWARNSD<br>VKEVDDTAMAFRLLRLHGYSVSPDVFKNFEKDGEFFAFVGQSNQA<br>VTGMYNLNRASQISFPGEDVLHRAGPFSYEFLRRKQAEGALRDKWI<br>ISKDLPGEVVYTLDFPWYGNLPRVEARDYLEQYGGGDDVWIGKTL<br>YRMPLVNNDVYLELARMDFNHCQALHQLEWQGLKKWYTENRLM<br>DFGVAQEDALRAYFLAAASVYEPCRAAERLAWARAAILANAVSTHL<br>RNSPSFRERLEHSLRCRPSEETDGSWFNSSSGSDAVLVKAVLRLTDSL<br>AREAQPIHGGDPEDIHKLLRSAWAEWVREKADAADSVCNGSSAVE<br>QEGSRMVHDKQTCLLLARMIEISAGRAAGEAASEDGDRRIIQLTGSI<br>CDSLKQKMLVSQDPEKNEEMMSHVDDELKLRIREFVQYLLRLGEK<br>KTGSSETRQTFLSIVKSCYYAAHCPPHVVDRHISRVIFEPVSAAK

**Zm00001d032961**	MKLLSPAAAPSSSPLFPPRIVEAAARQSGPCRIRIRIRGKAAAAGGGG<br>GAGATGPRGSLRLAGWWRAQQQAPATATTQQPDNVSSAKVFQTS<br>RVETESEIAKWPGKPQDLEDEHQAEEAELQPLIDQVRAMLRSMNDG<br>DTSASAYDTAWVAMVPKVGGDGGAQPQFPATVRWIVDHQLPDGSW<br>GDSALFSAYDRMINTLACVVALTKWSLEPARCEAGLSFLHENMWRL<br>AEEEAESMPIGFEIAFPSLIQTARDLGVVDFPYGHPALQSIYANREVK<br>LKRIPRDMMHRVPTSILHSLEGMPDLDWPRLLNLQSCDGSFLFSPSA<br>TAYALMQTGDKKCFEYIDRIVKKFNGGVPNVYPVDLFEHIWVVDRL<br>ERLGISRYFQREIEQCMDYVNRHWTEDGICWARKSNVKDVDDTAM<br>AFRLLRLHGYNVSPSVFKNFEKDGEFFCFVGQSTQAVTGMYNLNRA<br>SQISFQGEDVLHRARVFSYEFLRQREEQGMIRDKWIVAKDLPGEVQ<br>YTLDFPWYASLPRVEARTYLDQYGGKDDVWIGKTLYRMPLVNNDT<br>YLELAIRDFNHCQALHQLECNGLQTWYKDNCLDAFGVEPQDVLRS<br>YFLAAACIFEPSRAAERLAWARTSMIANAISTHLRDISEDKKRLECFV<br>HCLYEENDVSWLKRNPNDVILERALRRLINLLAQEALPIHEGQRFIH<br>SLLSLAWTEWMLQKANKEENKYHKCSGIEPQYMVHDRQTYLLLVQ<br>VIEICAGRIGEAVSMINNKDNDWFIQLTCATCDSLNHRMLLSQDTMK<br>NEARINWIEKEIELNMQELAQSLLLRCDEKTSNKKTKKTLWDVLRS<br>LYYATHSPQHMIDRHVSRVIFEPV
**Zm00001d048867**	MIEAIRAALGSTGDGEINISAYDTAWVALVKSLNGDEGPQFPSCIDWI<br>AQNQLPDGSWGDGIFFLVQDRIISTLACIIALKSWKIHDDACKKGTL<br>STALSNNNKPSYVLYRATYIYVTHEEYILLNRHWSEEGLAFTKGCRV<br>KDIDDTAMGFRLLRLHGYHVSPCVFKRFENGDGQFVCYARQSNQS<br>VSAMYNLYRAADQGSFPGDDPVLQRAKSYSRAFLRERRASDQLND<br>KWIISEGLPSEVGYGLDFPWEACLPRIETRMYLEQYGGSHDVWIGK<br>VLYRMNMVSNDMYLKAAKADFGNFQRLCRLEWHDLKRWCDKIS<br>NIETYGVASRGALLRAYFLAAACIFEPSRAAERLAWARAAVLAEAIS<br>CCLLSNVDDACACNKTTAEWLVQEFTNGDDIADSYEYSPARRDDD<br>GPNSPAWGASSLAGVLRELVNLQASGNAAVAGCLRGAWIEWLMAW<br>TGKETEKASRAGDTALLLARTVEICSGRRCGTEKDLDLLLRDYSKL<br>QQLTRCICSRLATEAPALNGETMDKIDALDRMVGLEMRELAQCVFR<br>SGGSSVDRETRQTFLHVTKSYYYVALCSPETVEHHISKVLFEDVV
**Zm00001d048874**	MSPWQCMPSTATLLHVHQVTDATRRLPPIRFSHLNAPNPRPRPGCA<br>MGATKGEKLKTLGHDLLVPATPQHKDKQSGMPEMIEAIRVALRSMG<br>DEFPSCIDWIAQNQLPDGSWGHDIFFLAQDRIINTLACVIALKSWKIH<br>DDACTKGLSFITENMWRLTRDDENWALSGFEIIFPMLLEKAKHLGID<br>IPLDDPMLEAIRAKRELKLNKIPREALHAEPTTFLLSIEGMPGLDWK<br>RLRKLQCPDGSYMSSPAPTAYALMQTGDAKCFEFLDKLIDKFNGGV<br>PFVYPMEMFGRLWAVDRLERLGISGYFKSEIEDYLDYVYRHWSEEG<br>LAYTKGCLVKDIDDTAMGFRLLRLHGYDHVSPCVFKRFENGDGQF<br>VCYARQSSQSVSAMYNLYRAADQASFPGDDDDHVLRRARSYSRAF<br>LRQRRASGQLNDKWIISEGLPDEVGYGLDFPWGASLPRIETRMYLE<br>QYGGSRDVWIGKVLYRMNMVSNDMYLEVAKVDFSNFQRLCRLEW<br>HDLKRWCDKSDLETYGVAPGGALRAYFLAAACIFEPGQAAERLAW

	ARAAVLAKAISCCLLSNNDTCACNKTTAEWLVREFTNGDNVAGYY
	YDYNPARRDDDEPNSPAWGASSLAGVLRELVHLQASGNAAVAECLR
	GAWMEWLMAWTEKETEEASHAGDTALLLARTVEICSGRLRGTEQD
	LELRLPDYSKLQQLTRCICSRLATEAPALIESNVLKQNGETMDKVDA
	LDRMVGLEMRELAQCVFRSGGSSVDRETRQTFLHVTKSYYYVALC
	SPETLEHHISKVLFEDVV
**Zm00001d041082**	MTPLRYAASPHLQPPFLGYKSSNVDGRSRTRQQAMIAERSSSSSTSG
	RGGQLEDRIREQLLNAELSPSSYDTAWAAMVPAPGSLAASRYPQCV
	DWILRNQRSDGSWGLDPSLRKDALSSTLACVLALRTWGVGEEHVG
	KGLRFIGHNSTSVTDCDKCDTPAGFNVIFPGMLERGIGMGLEIPLAH
	ADLDAILRLRDTELKSIAASGRSKAYMSYVAEGLGDLLDWDQAAM
	AYQRKNGSFFNSPATTAAAAIHNGYNKRAIGYLDALISESGSSSSVPA
	VYPRKVHSQLRMVDTLERMGISPSFSYEINAILDMTYSSWLRNDDE
	VMLDMATCAMAFRLLRMHGYHVSSDALAQFSEESSFHDSIEAHLN
	GTEALLELYKASQVQILEEEPVLESIFSWSGKLLRRKQCSDSDDPAE
	VEHVLKFPSYATLDRLEHRRNIERFKTGGFQMLKSAFRVSHADEEIL
	TLATDGFHSSQAVYQQEIEQLNRWVKEVRLPELEFARVLPLPILFSAA
	ATMFPAELSEARIAWSKSSVLTTIVDDLFDTGDSMEELENLVTLFDL
	WDAHQEAGFYSERVEIVFRAVYDTCKHLVGKAAAVQNRGVMDHIA
	DLWVDVVRAMMPEAEWRMSGRVPSMEEYLPIGEVSFAIGPIVPMA
	AYLVGPELPEDVVGSAEYEEMLSLVNLSGRLLNDLQTYERERKQGK
	INSVLLLADPSYCGSVEAATREVRSVIEASRRKLLRLVLRDGAAVPRP
	AGQLFWNMSKVLHLFYMDRDGYSSPLTNAIDEVLIKPLQMGRRRE
	EDQLPGRVRSNQN
**Zm00001d002349**	MPDVMAAALTGFSPRGVIPSRGRASLPGVARAYAERRLVAENTSLPN
	MHKEQLETIIRNQLRKPQLPPSSYDTAWVSMVPVRGSHQTPRFPQC
	VEWILQNQQDDGSWGVSQSDSSVSKDVLLSTLACVLALKRWNVG
	RENIWRGLHFIGRNFSVAMDEQFTSPIGFNFTFPGLLSLGIDMGLEFP
	VRQIDVCGILHRREMELKRLAVDSSFGRKAYMAFIPEGFGNMLDWD
	QVMKFQRKNGSLFSTPSTTAVALIHKYNDQALQYLNLLVNEFGSAVP
	AMYPSRVHCQLSMVDALEKMGISQRFVSEIESILDMAYNCWLQNDE
	ELMMDIATFAMAFRLLRMNGYDVSSDELSHVAGASTFHDSLQGYL
	NDTKSLLELYKTSKVTLSENDLILDRIGSWSGNLLKDKMCCSRVQK
	DSIFGEIEYAVNFPLYSTLERLEHKRNIEHFDAWGSLMLTTKSSSFRIN
	QEFLALAVEDFSFSQRVYRDELRHLDSWVKENKLDQLQFARQKLTY
	CYLSAAATVFSSELSDARISWAKNGVLTTVVDDFFDVGGSKEELEN
	LIALVEKWHGHHAVEFYSEQVKIVFSAIYTTVNHLGAMASAAQGRD
	LTNHLVEIWLDLLRSMMVEAEWQRCQYVPTVEEYMTNAVVSFALG
	PIVLPALYFVGQELLEHAVKDEEYDKLFRLVSTCGRLLNDYQSLERE
	GNQGKLNSVSLLVLHSGGSMSIEAAKKAMQKSIDVSRRDLLRLVLR
	KESAVPRPCKELFWKMCKILHLFYSQNDGFSSPKEMVSAVNAVINEP
	LKVQNSTTFLSSSSR
**Zm00001d002350**	MAKPLNLTPAFLSLMATSCRFSSGGHAHRFASSLPCGRTTPTPRTTSS
	STRPAAQVSPDRSKQRDDEAASETTIRQQLQQVDVLENTGISRHFAG

	EIKCILDRTYRCWLERHEEIMQDTTTCAMAFRILRMNGYDVSCDLL
	YHVVETSGIHDPLRGHLNDTRTLLELHKASTVSISEDESILDSIGLRSS
	TLLREQMESSGALQNPPLFREVEHALYHPFYATLDRLTHRWNIENFN
	TTEHQMLDTPYLSSRYTSRDILALGIRDFNSSQLTYQQELQHLKSWV
	KECRLDQLPFVRQNLAYFLLSAAGCMYSPELSEARTLCAKNGALIT
	VVDDFFDVGGSKEELENLVMLVELWDEHRKIEFYSEHVEIIFSSIYNS
	VNQYGAKASLLQDRNVTKHLVQIWLDLLKSMMTEVEWRLNKYVP
	TEEEYMTNASLTFALGPIVLPALYFVGPKIPESIVKDPEYDELFKLMS
	TCGRLLNDVQSFEREYKEGKLNIVSLLVLHGGSMSISDARRKLQKPI
	DTCRRDLLRLVLREESVVPRPCKELFWKMCKVCYFFYSRTDGFRSP
	VERAKEVNAVINEPLKLQENHTHMSVL
**Zm00001d002351**	MAMPVKLTPASLSLKAVCCRFSSGGHALRFGSSLPCWRRTPTQRSTS
	SSTTRPAAEVSSGKSKQHDQEASEATIRQQLQLVDVLENMGISRHFA
	AEIKCILDRTYRSWLQRHEEIMLDTMTCAMAFRILRLNGYNVSSDEL
	YHVVEASGLHNSLGGYLNDTRTLLELHKASTVSISEDESILDSIGSRS
	RTLLREQLESGGALRKPSLFKEVEHALDGPFYTTLDRLHHRWNIENF
	NIIEQHMLETPYLSNQHTSRDILALSIRDFSSSQFTYQQELQHLESWV
	KECRLDQLQFARQKLAYFYLSAAGTMFSPELSDARTLWAKNGVLTT
	IVDDFFDVAGSKEELENLVMLVEMWDEHHKVEFYSEQVEIIFSSIYD
	SVNQLGEKASLVQDRSITKHLVEIWLDLLKSMMTEVEWRLSKYVPT
	EKEYMINASLTFGLGPIVLPALYFVGPKISESIVKDPEYDELFKLMST
	CGRLLNDVQTFEREYNEGKLNSVSLLVLHGGSMSISDAKRKLQKPI
	DTCRRDLLSLVLREESVVPRPCKELFWKMCKVCYFFYSTTDGFSSQ
	VERAKEVDAVINEPLKLQGSHTLVSDV
**Zm00001d024514**	MSTVWCSSVLSATATSVPKNPSRSNPSNHLRFTFSNSSAKSSCHRFSS
	CRSNRPPPLYYWACIESRRRISCGVQTTAQSGTRVVERNASLEIEERH
	ARIRKQLLKPELSPSAYDTAWVAMVPSPGSPQAPCFPQCVQWILENQ
	LDNGSWGHNEVDSSADKKETILATLACVIALKKWDVGPAHITKGIQ
	FIARNFQTVTDEQIVVPIGFDFTFSGMFTLAINMGLQFPVRQTDIDGI
	LRLRENELKRYDGDKSTAIEAYCAYVAEGFENLLDWNDVMKFQAK
	NGSLFNSPSATAAALVANYDDKALQYLNLLVTQFGSAVPTVFPQNIH
	CQLSMVDTLESVGISRHFSAEIKAVLDMVYSLWLQKEEEIMLDAET
	CAMAFRILQMNGYDVSTDWLSHIAEASNFHNSLQGYLCDTKTLLE
	MYKASKVILSERDLTLENIGCWTGSLLREKLCSDGAQRVPILEEVEY
	ALKFPHYAIVDPLNNRRSIEHLDARGSQTLKTKYLPCHVSQDILALA
	VEDFCFSQSIYQDELQNIISWEKENRMDQLQFVRQRLAYCYLAAAT
	TISPHELSDARVACAKSIMLTVVVDDFFDVGGSKEEQENLIELVENW
	DEHHKVEFCSEKVEIVFYAVYNTVNQLGSMASAVQKRDVTKHLAE
	TWLKVMLCMLTEADWQRRQFVPTVEEYMANAVSSLALAVIILPAQ
	YFLGETLSDYMVKDHEYSNLSELMFTCSRLLNDIRSVEREFEAGKL
	NSVSLLALHSGGSMSIEAAKKETHGSLESYRRGLVALVRRQDSVVP
	RSCKELFWKLCKTIHLFYFQIDGFTSPKEMVGAVNQVINEPLELQSS
	NTHV
**Zm00001d032858**	MASLSFASSHASLFCCQQSSSAIILRPAGALLRLSRRQPSSHTISTTDQ

	LFPRRSRMPRNVDTHAAAERNSPSTMSSLEAVDELETNGDSAVVVV
	REQQQQQHLLMGATDDGLPPSPYDTAWVAMVPAPGNPLVPRFPQC
	VDWILQNQRSDGSWGPDGGSGDHPSSPLGKDALMSTLACVLALKT
	WDAGEEHVRKGLSFVGNNSPSCVMTGDERDAPVGFSVIFPGMLAR
	AIDMGLDIPMMTQANVDAFIRLRDTELNRMAATTGSKAFMSYVAE
	GLGDVLDWDEAAMVYQRQNGSFFNSPATTAAAAIHGNNDRALRYL
	DSLVNMFGSSVPTVYPRSTYSRLHMVDTLQKMGLSRSFVSEINEML
	DMTYRSWLANDDEEMMLDMSTCAMAFRLLRMHGYDVSSDGLAQ
	FSSESSFRDSVHGQANDTEALLELYKASQIQITEDELVLVDIRSWSAK
	LLKEQLGSDKVSRSVDAQEVQQVLKFPFYTTLDRLEHRRHIEQFKA
	GGFHMLKSAYRFCKEDEELVSLAVQGFHSSQALYQQELQFLTRWAK
	EARLHDLEFARIMPMNTFFPNAALMYAPELSEARILCTKNCMLATAV
	DDLFDVGGSREEMENLVRLIDMWDEHEEVGFCSERVEILFRAIYDTS
	KELAAKAMAVQNRSVINHVAELWADLVRAMMTEAEWSMRGHVPS
	SMEEYMQVAETSFALGPIVLMPLYLIGPELPEAVVRCPEYKQLFHHM
	NVCGRLLNDLQSYEREGKQGKINSVLLVAPRHGGSIEAAKSEVRRAI
	EASRRELLRMLVAEADATVPRPFRQEFWNMCKMVHLFYMEDDCYS
	SPKELVHAANMVVFDPLRVREL
**Zm00001d049957**	MASFSYGGAPPPSHSGLRHYPTVHRPGLLPATAACTRGGAELLRRQ
	SRATTPRTKLAEKMVREKLLMGLPPPSSYDTAWLAMVPAPGSARAS
	RFPQCVEWIMQNQRSDGSWGLQGRQGDDPSLLIKDALSSTLACVL
	ALKTWGVGDEHVGKGLRFIGHNLSSVTTYGDVGSRPVGFNVIFPG
	MLARCIGMGVEMPLAPADVDGIFRLRDKELPRWLDVDFGTSTIIKA
	FRAYLAEGLGNWDEAMAYQRKNGSLFNSPATTAAAAIHSYDRGAV
	QYLDSLVNLFGSSVPAMYPRILYARLRMVDALEKTGISRSFSSEINGI
	LDMTYASWLGNDEEIMLDMTTCAMAFRLLRMHGYDVSSDASLGQ
	FSEESSFHDSIQGCLGDTEALLELHKASQVQIREQETAILRSIGSWSA
	KLLEQQLRSNKVSRNIDPAEVDYALKHPFYTTLDRLEHRRNIEHFKT
	TAIQVLKSAYRACVPAGEEIAALAAADFHASQTVYQEELQCLESWV
	KELRLQELEFARIMPLICFFPPAATLFPCEQHEARMAMSKHNILLTVV
	DDLFDVGGSREEMENLVKLVEMWDAHNRVGFCSERVEIVFRAIYDT
	SNHLAAKAAAVQKRNIIHHLAELWANTVRAMMAEANWAMTGHLP
	TFEEYMAAGEPSFGLGPIVLASLYLVGPELPEDVVTSQEYNEMFRH
	MNVCGRLLNDLQSYEREKEQGKVNSVLLLLALEEEGGSIEAVKREV
	RRAIEASRIQLLRLVLRNESEVPWPCRQVFWNICKVVHQFYVHVDG
	YASAKQMMHSCTPGTRWCITRCRSHARRPTYNKFSQLSLGVGVHC
**Zm00001d015053**	MYSLPGATMSAAPASIISSSSFVEPLLLAAASPAAAAAAANSHHQVR
	QRGHLVRTLAASSSSNTLLRSDFDLQEGLTTDVKRMLRQRQKKSGG
	GREMLVTIDNLKRLCIDHYFEEEIEGAMATGACTRLLHSDDLFDATL
	AFRLLREAGHDVSAKDDVLRRFIDGASGDFKLSLSNDVRGLLSLHD
	MSHLDVGGEAALLHRAKEFSSRHLASAVRYLDDPSLAEYVRQSLD
	HPYHLSLTQYKARHHLRYLQSLPSRDAAVERLAVAEFQLNKSLHQG
	EMREIKRWWMDLGLAEEIPVVRDQVMKWYMWSMAALQGSSFSR
	YRVEITKIISLVYVVDDIFDLVGTLEELSAFTEAVKMWDTVAADSLPS

	CMRSCYKALHTVTNEIAEIAQKEHGSNHVNRLRKAWAVLFDGFMVEARWLATDQVPTAEDYLRNGVITSGVPLTFMHIFSMLGYDDPSTEEEEEAIIDHMPSIISCPAKILRLWDDMGSAEDEAQEGFDGSYRDFYLMENPSRSPGEAEAHMRGLIAREWEVLNRECFCRRTFPSNLVQVCLNTARMVSVMYSYNKEQRLPVLEDYAAMMLVL
**Zm00001d015054**	MYSLPGATMSAAPARVISSSSSSSFVEPLLLAAASSAAANSHHQVRQRGHLVRTLAASSSSNTLLRSDFDLQEGLTTDVKRMLRQRQKKSGGGREMLVTIDNLKRLCIDHFFEEEIEGAMATGACTRLLHSDDLFDATLAFRLLREAGHDVSAKEDVLRRFIDGVSGDFKLSLNNDVRGLLGLHDMSHLDVGGEEAALLHRAKEFSSSHLASAVRYQDNPSLAEYVRQSLDHPYHLSLTQYKARHHLRYLQSLPSSCRDAAVERLAVAEFQLNKSLHQREMREIKRWWMDLGLAEEIPVVRDQVMKWYMWSMAALQGSSFSRYRVYVVDDIFDLVGTLEELSAFTEAVKMWDTAAADSLPSCMRSCYKALHTVTNEIAEIAHKEHGSNPINRLRKAWVVLFDGFMVEARWLATDQVPTAEDYLRNGVVTSGVPLTFLHIFSMLGYDDPSTEEEEEAIIDHMPSIISCPAKILRLWDDMGSAEDEAQEGFDGSYRDFYLMENPSRSPGEAEAHMRGLIAREWVELNRECFCRRTFPSDIAQVCLNTARMVSVMYSYNKEQRLLVLEDYAAMMLVL
**Zm00001d053916**	MLATPHHINLEGLRTPRCISLTCILAPARRIGTSMSERCIFSFSTLASPAADGNNSRRGGRHRGDSIVTRSSAAANTLPLLRNDDFDLREGLTDVRNVLRQRQKSGCSSRDMMVTVDNLKRLCIDHYFQEEIERAMGACCMGLIHSDDLFDATLAFRLLREAGHGVSAADDVLRRFTDDDDSGGEFRLDLSKDIRGLLSLHDMSHLDMGGEELLCKARVFSRHHLASAVRYLEPSLGEYVRQSLDHPYHLSLTQYKARHHLAYLQSLPVRDTALERLVAAEFQLNRSLHQKEMQAIKRWWMELGLVQEIPVVRDQVLKWYMWSMTALQGRSFSRHRVQITKIIALVYVVDDIFDLVATLDELSLFAEAVKMWNSTAAESLPSYMRSCYKALYTVTNEIADTAEKEHGLNHVNHLRKAWAALFDGFMVEARWLATDKVPTAEDYLTNGVVTSGVPLTFVHIFIMLGCDDDQSTQALVDHMPSVISCPAKILRLWDDMGSAEDEAQEGFDGSYRDFYLMENPRCSRSDAEAHMRSLIAREWEELNRECLCKRTFSSSFTHVCLNAARMISVMYSYNKEQRLLVLEDYARMLIL
**Zm00001d053918**	MPQGTLPCYECRVLKWYMWSMTALQGSSFSRFRVEMTKIISLVYVVDDIFDFVGTLEELSLFTEAVKMWDTVAADSLPSYMRSCYNALYTITNEIAEMAEKKHGLNSINHLRKAWATLFDGFMVEARWLAIDQVPTAEDYLRNGVVTSGVPLTFVHIFTLMGCEQSIEALIDQMPSVISCPAKILRLQDDMGSAKDEAQEGFDGSYRDFYLKENPRCNRNDAEAHMRSLIAREWEELNRECLYTRTFSSNCTRVCLNTARMIGVMYSYNKEQRLLVLEDYAKMLIL
