# Supplementary material for: Identification and functional analysis of ZmDLS associated with the response to biotic stress in maize
Source: Front Plant Sci. 2023 Jul 20;14:1162826. doi: 10.3389/fpls.2023.1162826 (PMC10399692; doi:10.3389/fpls.2023.1162826)
Supplement: Supplementary Table 8 — Summary of qRT-PCR on data [file DataSheet_1.zip › Supplementary Table 3.PDF]

Table S3. Basic physical and chemical properties of 43 ZmTPSs

Gene name	Aliphatic index	Grand average of hydropathicity	stable yes/no
Zm00001d000337	84.19	-0.35	no
Zm00001d004484	90.74	-0.261	yes
Zm00001d004509	93.38	-0.145	yes
Zm00001d024207	90.86	-0.261	no
Zm00001d024208	93.89	-0.227	yes
Zm00001d024210	90.49	-0.287	no
Zm00001d024211	90.69	-0.269	yes
Zm00001d024234	89.24	-0.322	no
Zm00001d024359	95.44	-0.165	no
Zm00001d024477	86.46	-0.29	no
Zm00001d024478	84.53	-0.342	no
Zm00001d024479	93.89	-0.227	yes
Zm00001d024480	99.47	0.059	no
Zm00001d024481	84.53	-0.337	no
Zm00001d024486	89.68	-0.243	yes
Zm00001d024667	97.52	-0.169	no
Zm00001d024669	85.36	-0.379	no
Zm00001d029139	82.73	-0.3	no
Zm00001d029195	89.89	-0.268	yes
Zm00001d029523	85.22	-0.403	no
Zm00001d032230	88.44	-0.248	yes
Zm00001d035682	89.67	-0.296	no
Zm00001d037092	82.97	-0.286	no
Zm00001d045054	86.67	-0.156	no
Zm00001d047440	81.93	-0.193	no
Zm00001d004279	89.37	-0.326	no
Zm00001d018611	86.47	-0.16	no
Zm00001d024512	84.93	-0.241	no
Zm00001d029648	81.85	-0.343	no
Zm00001d032961	82.87	-0.363	no
Zm00001d048867	84.52	-0.222	no
Zm00001d048874	81.58	-0.308	no
Zm00001d041082	87.34	-0.241	no
Zm00001d002349	89.7	-0.159	no
Zm00001d002350	87.9	-0.328	no
Zm00001d002351	89.86	-0.27	no
Zm00001d024514	88.69	-0.163	no
Zm00001d032858	81	-0.226	no
Zm00001d049957	86.26	-0.166	no
Zm00001d015053	88.54	-0.197	no

Zm00001d015054	86.2	-0.24	no
Zm00001d053916	89.15	-0.222	no
Zm00001d053918	85.59	-0.111	no
