# Supplementary material for: Identification and functional analysis of ZmDLS associated with the response to biotic stress in maize
Source: Front Plant Sci. 2023 Jul 20;14:1162826. doi: 10.3389/fpls.2023.1162826 (PMC10399692; doi:10.3389/fpls.2023.1162826)
Supplement: Supplementary Table 8 — Summary of qRT-PCR on data [file DataSheet_1.zip › Supplementary Table 4.PDF]

Table S4. Analysis of *cis*-acting elements in *ZmDLS* promoter

Site Name	Organism	Position	Matrix score	Sequence
GARE-motif	*Brassica oleracea*	1854	7	TCTGTTG
plant_AP-2-like	*Oryza sativa*	1783	8	CGACCAGG
GT1-motif	*Arabidopsis thaliana/Avena sativa*	220,541,1899	6,7	GGTTAA/GGTTAAT
TATA	*Arabidopsis thaliana*	6,397	8	TATAAAAT
ATC-motif	*Spinacia oleracea*	711	8	AGTAATCT
Myb-binding site	*Nicotiana tabacum*	594,725,1854	6	CTGTTG/CAACAG
MYB	*Arabidopsis thaliana*	221,540,1212, 608, 638, 356	6	TAACCA/CAACCA/ TAACTG
DRE-motif	*Arabidopsis thaliana*	1756	6	GCCGAC
Box 4	*Petroselinum crispum*	331	6	ATTAAT
AuxRR-core	*Nicotiana tabacum*	1918	7	GGTCCAT
STRE	*Arabidopsis thaliana*	461	5	AGGGG
AAGAA-motif	*Avena sativa*	1331	9	GTAAAGAAA
CAAT-box	*Nicotiana glutinosa/Pisum sativum/Arabidopsis thaliana*	87,120,228,312,849 895,1146,1589,1608 1733,1737,106,312 849,1248,1588,1607 259	4,5,5,8	CAAT/CAAAT/CCAAT/ CCCAATTT
G-Box	*Arabidopsis thaliana*	1631	6	CACGTG
CAT-box	*Arabidopsis thaliana*	1598	6	GCCACT
TGA-element	*Brassica oleracea*	946	6	AACGAC
ATCT-motif	*Pisum sativum*	714,1294,1604	9	AATCTAATCC
TATA-box	*Arabidopsis thaliana*	10,59,231,300,308 320,362,397,678,887,939	4	TATA
GCN4_motif	*Oryza sativa*	1705	7	TGAGTCA
TCT-motif	*Arabidopsis thaliana*	183	6	TCTTAC
ABRE	*Arabidopsis thaliana*	1631,1632	5	ACGTG
WRE3	*Pisum sativum*	100	6	CCACCT
LTR	*Hordeum vulgare*	1963	6	CCGAAA
MYC	*Arabidopsis thaliana*	1566,1589,1701	6	CATGTG
O2-site	*Zea mays*	1466,1907	9	GATGATGTGG
ARE	*Zea mays*	283,1558	6	AAACCA
AE-box	*Arabidopsis thaliana*	1388	8	AGAAACAA
