# Supplementary material for: Identification and functional analysis of ZmDLS associated with the response to biotic stress in maize
Source: Front Plant Sci. 2023 Jul 20;14:1162826. doi: 10.3389/fpls.2023.1162826 (PMC10399692; doi:10.3389/fpls.2023.1162826)
Supplement: Supplementary Table 8 — Summary of qRT-PCR on data [file DataSheet_1.zip › Supplementary Table 5.PDF]

Table S5. Primer names and sequences

Prime name	Primer names and sequences
q-*ZmActin1*-S	CTGGTGCCCTCTCTCCATATGG
q-*ZmActin1*-AS	CAACACTGACACGACTCATGACA
q-*Zm00001d032230*-S	CCCTGCTCTATTCCCGGTTG
q-*Zm00001d032230*-AS	TGGGTCTCCAGTGTCGTTTG
q-*Zm00001d045054*-S	GCTCGCGTGAAGATACAGGA
q-*Zm00001d045054*-AS	AGGCTCCACAAACAACGAGT
q-*Zm00001d024486*-S	CGTACGTTAGGACTCACGGG
q-*Zm00001d024486*-AS	CCTCGTTTCGTCCAGCTTCT
q-*Zm00001d004279*-S	AGTTGCCAGTATTCTATGC
q-*Zm00001d004279*-AS	ACCATCTCCACCTTGATAA
q-*Zm00001d002351*-S	ACGACGGATGGGTTTAGCTC
q-*Zm00001d002351*-AS	TCACACATCAGACACCAGCG
q-*Zm00001d002350*-S	CACAGGTGGAACATTGAA
q-*Zm00001d002350*-AS	AGAATATCTCTACTGGTATATCG
q-*Zm00001d053916*-S	TAGATGCAGCCGTAGCGATG
q-*Zm00001d053916*-AS	CAAGCAGACGTGTGTGAAGC
q-*Zm00001d015053*-S	TGGGTCGTACAGGGACTTCT
q-*Zm00001d015053*-AS	GGAAGCCTCTGCTCCTTGTT
q-*Zm00001d015054*-S	CAGCGTCATGTACTCGTA
q-*Zm00001d015054*-AS	TCAAAGCACCAACATCATC
Zm00001d045054-S	ATGGTAGCAGCAGCCAGCACCA
Zm00001d045054-AS	GGCTATGGAAACAGGCTCCACAAAC
Zm00001d045054-pMAL-S	CGGAATTCATGGTAGCAGCAGCCAGC
Zm00001d045054-pMAL-AS	ACGCGTCGACGGCTATGGAAACAGG
Zm00001d045054-1305-S	aagtccggagctagctctagaATGGTAGCAGCAGCCAGCACCA
Zm00001d045054-1305-AS	gcccttgctcaccatggatccGGCTATGGAAACAGGCTCCACAAAC
q-*ZmMYB76*-S	TTCCAGTCATTGGGATGGGC
q-*ZmMYB76*-AS	TGCATGTGGTGCCTCAATCT
q-*ZmMYB91*-S	TGCTGTTGGTGGTCACTGTT
q-*ZmMYB91*-AS	GTCGATCCTCCACAGCGAAA
q-*ZmMYB97*-S	CGCACCGATATCTCCATCAAG
q-*ZmMYB97*-AS	ATGAGATCGGGAATCGGCAC
q-*ZmMYB101*-S	CTGCTCTGGCTCTCCTAGTC
q-*ZmMYB101*-AS	CTCGGTCAGAGCGGACGAT
q-*ZmMYB104*-S	ACGAACAGTGCACCAACAAC
q-*ZmMYB104*-AS	GACCACTTGAGCTCGTCCAG
q-*ZmMYB119*-S	GCAGTCTCTATCAGCGACCC
q-*ZmMYB119*-AS	GGTTGTTGGCTCTTCCCAGA
ZmMYB76-1305-S	aagtccggagctagctctagaATGTCACCACAAGAAGAGCGC
ZmMYB76-1305-AS	gcccttgctcaccatggatccCATCATGATTTCTTGATCTCCC
ZmMYB101-1305-S	aagtccggagctagctctagaATGCGCATGTGTCGGCGG

ZmMYB101-1305-AS	gcccttgctcaccatggatccGGAGGAGGAGTCGAGCA
ZmDLS-PRO-S	GGTTCATACGAAGAGCATGTATTAGTG
ZmDLS-PRO-AS	CTGTGTAGCTCTCAACCAAGCTACC
ZmDLS-AbAi-S	aattcgagctcggtacccgggGGTTCATACGAAGAGCATGTATTAGTG
ZmDLS-AbAi-AS	atacagagcacatgcctcgagCTGTGTAGCTCTCAACCAAGCTACC
ZmMYB76-ADT7-S	gccatggaggccagtgaattcATGTCACCACAAGAAGAGCGC
ZmMYB76-ADT7-AS	cagctcgagctcgatggatccTCACATCATGATTTCTTGATCTCCC
ZmMYB101-ADT7-S	gccatggaggccagtgaattcATGCGCATGTGTCGGCGG
ZmMYB101-ADT7-AS	cagctcgagctcgatggatccTCAGGAGGAGGAGTCGAGCA
ZmDLS-0800-S	cttgatatcgaattcctgcagGGTTCATACGAAGAGCATGTATTAGTG
ZmDLS-0800-AS	cgctctagaactagtggatccCTGTGTAGCTCTCAACCAAGCTACC
ZmMYB76-62sk-S	cgctctagaactagtggatccATGTCACCACAAGAAGAGCGC
ZmMYB76-62sk-AS	gataagcttgatatcgaattcTCACATCATGATTTCTTGATCTCCC
ZmMYB101-62sk-S	cgctctagaactagtggatccATGCGCATGTGTCGGCGG
ZmMYB101-62sk-AS	gataagcttgatatcgaattcTCAGGAGGAGGAGTCGAGCA
