# Supplementary material for: Identification and functional analysis of ZmDLS associated with the response to biotic stress in maize
Source: Front Plant Sci. 2023 Jul 20;14:1162826. doi: 10.3389/fpls.2023.1162826 (PMC10399692; doi:10.3389/fpls.2023.1162826)
Supplement: Supplementary Table 8 — Summary of qRT-PCR on data [file DataSheet_1.zip › Supplementary Table 6.PDF]

Table S6. Formula for protoplast preparation

Recipe for the enzymatic solution

Stock Solutions	Final Concentration
Cellulase R-10	1.5%
Macerozyme R-10	0.4%
D-mannitol	0.4 M
KCl	20 mM
MES (pH=5.7)	20 mM
55°C 10 min, leave to cool at room temperature	
$CaCl_2$	10 mM
β-mercaptoethanol	5 mM
BSA	0.1%

Recipe for the W5 solution

Stock Solutions	Final Concentration
NaCl	154 mM
$CaCl_2$	125 mM
KCl	5 mM
MES (pH=5.7)	2 mM

Recipe for the MMg solution

Stock Solutions	Final Concentration
Mannitol	0.4 M
$MgCl_2$	15 mM
MES (pH=5.7)	4 mM
