# Supplementary material for: Identification and functional analysis of ZmDLS associated with the response to biotic stress in maize
Source: Front Plant Sci. 2023 Jul 20;14:1162826. doi: 10.3389/fpls.2023.1162826 (PMC10399692; doi:10.3389/fpls.2023.1162826)
Supplement: Supplementary Table 8 — Summary of qRT-PCR on data [file DataSheet_1.zip › Supplementary Table 7.PDF]

Table S7. Correlation coefficients and *p* values of *ZmDLS* and *ZmMYBs* ($r>0.6$, $p<0.01$)

Gene1	Gene2	Correlation	*p* value
ZmDLS	*ZmMYB091*	0.609935301	0.002000134
ZmDLS	*ZmMYB119*	0.711900584	0.000139025
ZmDLS	*ZmMYB104*	0.751980571	3.51E-05
ZmDLS	*ZmMYB101*	0.793412401	6.28E-06
ZmDLS	*ZmMYB076*	0.800030556	4.60E-06
ZmDLS	*ZmMYB097*	0.996319635	6.78E-24
